# Lipid-Lowering Polyketides from the Fungus *Penicillium Steckii* HDN13-279

**DOI:** 10.3390/md16010025

**Published:** 2018-01-12

**Authors:** Guihong Yu, Shuai Wang, Lu Wang, Qian Che, Tianjiao Zhu, Guojian Zhang, Qianqun Gu, Peng Guo, Dehai Li

**Affiliations:** 1 Key Laboratory of Marine Drugs, Chinese Ministry of Education, School of Medicine and Pharmacy, Ocean University of China, Qingdao 266003, China; Yuguihong1990@126.com (G.Y.); wanglu784940819@163.com (L.W.); cheqian064@ouc.edu.cn (Q.C.); zhutj@ouc.edu.cn (T.Z.); zhangguojian@ouc.edu.cn (G.Z.); guqianq@ouc.edu.cn (Q.G.); 2 Institute of Medicinal Plant Development, Chinese Academy of Medical Sciences & Peking Union Medical College, Beijing 100193, China; zhuizhirun@163.com; 3 Laboratory for Marine Drugs and Bioproducts of Qingdao National Laboratory for Marine Science and Technology, Qingdao 266237, China

**Keywords:** *Penicillium steckii*, tanzawaic acid derivatives, lipid-lowering activity

## Abstract

Seven new polyketides, named tanzawaic acids R–X (**1**–**6**, **11**), along with seven known analogues (**7**–**10** and **12**–**14**), were isolated from *Penicillium steckii* HDN13-279. Their structures, including the absolute configurations, were elucidated by NMR, MS, X-ray diffraction, circular dichroism (CD) analyses and chemical derivatization. Five compounds (**2**, **3**, **6**, **10** and **12**) significantly decreased the oleic acid (OA)-elicited lipid accumulation in HepG2 liver cells at the concentration of 10 μM, among which, four compounds (**3**, **6**, **10** and **12**) significantly decreased intracellular total cholesterol (TC) levels and three Compounds (**3**, **6**, and **10**) significantly decreased intracellular triglyceride (TG) levels. Moreover, the TG-lowering capacities of compounds **6** and **10** were comparable with those of simvastatin, with the TG levels being nearly equal to blank control. This is the first report on the lipid-lowering activity of tanzawaic acid derivatives.

## 1. Introduction

Metabolic syndrome (MetS), including obesity, insulin resistance (IR), dyslipidemia and hypertension, have long been a worldwide problem [1,2,3]. Dyslipidemia, as one of the most common causes of MetS, can further result in atherosclerosis, myocardial infarction and cerebrovascular diseases, seriously threatening human life [4,5,6]. Due to the current undesirable side-effects of lipid-regulating drugs, it is urgent to find new classes of bioactive compounds with lipid-lowering capacity and a safer profile [7,8,9,10]. Famous drugs for the treatment of dyslipidemia are lovastatin analogues, reported to be produced by various fungal species, including *Aspergillus* spp., *Penicillium citrinum*, *Pleurotus* spp., and *Monascus ruber*, thus, suggesting fungi as promising sources for the discovery of new drug leads against MetS [11,12].

In our ongoing search for new bioactive metabolites from natural sources [13,14], *Penicillium steckii* HDN13-279 was selected for investigation due to the interesting HPLC–UV profile of its ethyl acetate extract. This species, widely distributed through marine and terrestrial environments, has been reported as an important producer of tanzawaic acids [15,16,17,18]. Tanzawaic acid derivatives are a class of polyketides characterized by a core structure with a poly-hydrogenated naphthalene ring and a penta-2,4-dienoic acid side chain. Several bioactivities have been reported for these compounds, namely anticoccidial, cytotoxic, anti-inflammatory, and capacity to inhibit protein tyrosine phosphatase 1B (PTP1B) and superoxide anion production [18,19,20,21,22,23,24]. In the present study, we report the isolation and structure elucidation of seven new polyketides and seven analogues isolated from *P. steckii* HDN13-279. The capacities of the isolated compounds to decrease oleic acid (OA)-elicited lipid accumulation in HepG2 liver cells were also reported.

## 2. Results and Discussion

**Figure 1:**
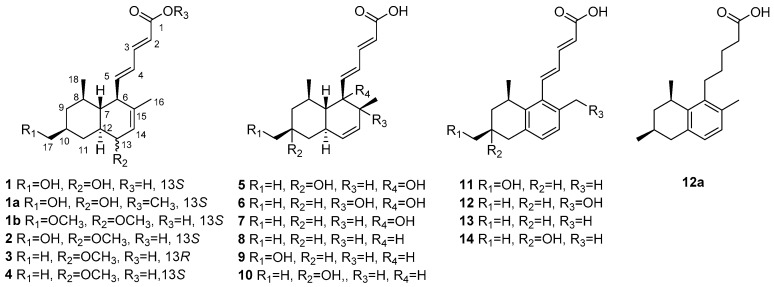
Structures of Compounds **1**–**14**, **1a**, **1b** and **12a**.

The fungal strain *P. steckii* HDN13-279 was fermented (45 L) under shaking conditions at 28 °C for 9 days. The EtOAc extract (40 g) was fractionated by silica gel vacuum liquid chromatography (VLC), C-18 ODS column chromatography, Sephadex LH-20 column chromatography, ODS MPLC, and finally HPLC to yield Compounds **1**–**14** (Figure 1).

**Figure 2:**
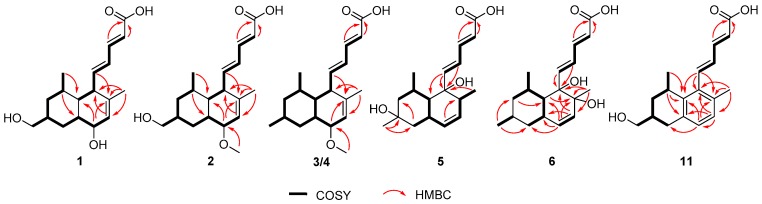
Key COSY and HMBC correlations of Compounds **1**–**6**, and **11**.

Tanzawaic acid R (**1**) was obtained as a pale yellow oil with the molecular formula C_18_H_26_O_4_ analyzed by HRESIMS. The 1D NMR data (Table 1) indicated the presence of two methyls, three methylenes (with one oxygenized), 11 methines (including five sp^2^ methines and one oxymethine), and two non-protonated carbons (including one carbonyl). The planar structure of Compound **1** was proved to be the same as tanzawaic acid H [25], supported by the similar 1D NMR and 2D NMR data (Figure 2). The clear differences in 1D NMR spectra (see Supplementary Materials), especially those at CH_2_-11 (δ_C_ 32.6; δ_H_ 1.66, 1.13 in **1** vs. δ_C_ 33.7; δ_H_ 2.32, 0.65 in tanzawaic acid H), CH-12 (δ_C_ 42.6, δ_H_ 1.29 in **1** vs. δ_C_ 47.3, δ_H_ 1.13 in tanzawaic acid H), CH-13 (δ_C_ 67.0, δ_H_ 3.83 in **1** vs. δ_C_ 72.4, δ 3.70 in tanzawaic acid H) and CH-14 (δ_C_ 125.5, δ_H_ 5.83 in **1** vs. δ_C_ 130.9, δ_H_ 5.55 in tanzawaic acid H) [25], suggested they have different stereochemistry.

**Figure 3:**
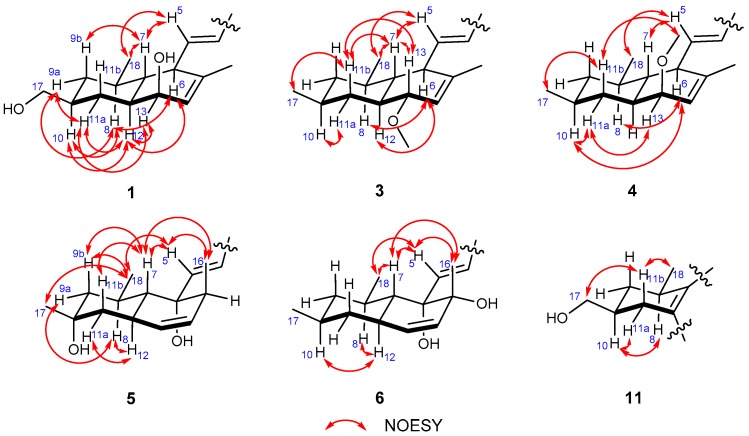
Key NOESY correlations of Compounds **1**, **3**–**6**, and **11**.

**Figure 4:**
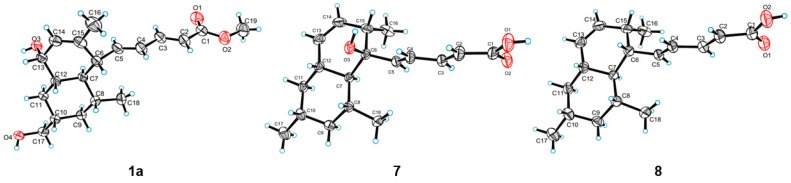
X-ray crystallographic structures of **1a**, **7**, and **8** (black, blue and red on behalf of the elements of C, H, O, respectively).

The relative configuration of **1** was assigned by NOESY spectroscopic data. The NOESY correlations from H-5 to H-7/H_3_-18, H-6 to H-8/H-12 indicated a *trans* fusion of the rings and placed H-7, Me-18 and the penta-2,4-dienoic acid moiety on the same side of the decalin ring, while H-6, H-8, H-12 on the other side. In addition, the NOESY correlations between H-9a (δ_H_ 1.69) and H-8/H-11a (δ_H_ 1.66), H-9b (δ_H_ 0.74) and H-7, H-10 and H-8/H-12, H-11a and H-12/H-13, H-12 and H-13 indicate that the hydroxymethyl at C-10 and the hydroxyl at C-13 were located at the same side as the penta-2,4-dienoic acid moiety (Figure 3). Thus, the NOESY data suggested that Compound **1** was a C-13 epimer of tanzawaic acid H. In a previous report [25], the absolute configuration of tanzawaic acid H was deduced based on the proposed biogenetic pathway. To make a solid evidence, we determined the absolute configuration of **1** by X-ray diffraction following chemical derivatization. Firstly, Compound **1** was esterified to generate the methyl ester (**1a**) and then the single crystal (CCDC 1537543) of **1a** was successfully obtained (Figure 4) with Flack parameter = 0.02 (11). Consequently, the absolute configuration of **1** was elucidated to be 6*R*, 7*R*, 8*R*, 10*S*, 12*S*, 13*S* (Figure 1).

**Figure 5:**
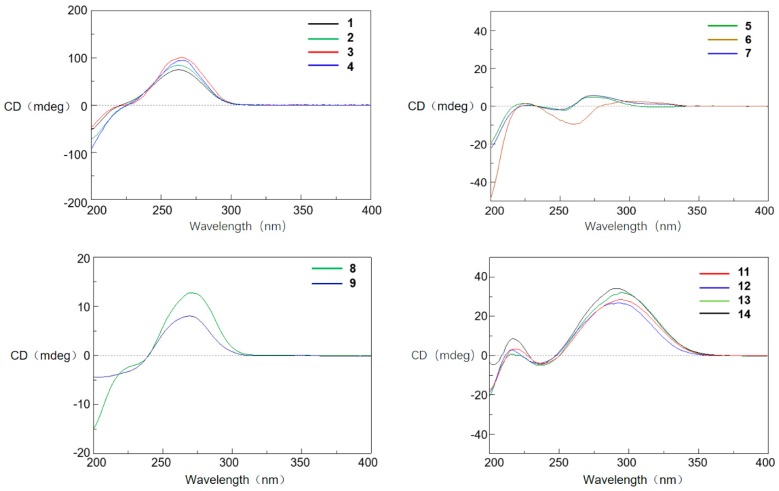
Experimental ECD spectra of Compounds **1**–**9** and **11**–**14**.

Tanzawaic acid S (**2**) was isolated as a pale yellow oil. The molecular formula of **2** was established as C_19_H_28_O_4_ according to the HRESIMS ions detected at *m*/*z* 319.1906 [M − H]^−^. The ^1^H NMR data of **2** was similar to those of **1** (Table 1), and the only difference was the existence of signal for an additional methoxy (δ_H_ 3.33). Further analysis of the 2D NMR spectra indicated the additional methoxy was attached at C-13 (δ_H_ 76.3) in **2** (Figure 2). The relative configuration of **2** cannot be completely assigned by NOESY data as the signals for H-7, H-8 and H-12 were overlapped. We further resorted to chemical derivatization. Both Compounds **1** and **2** were successfully transformed into Compound **1b** when methylated by NaH and CH_3_I in DMF solution, which suggested they share the same relative configuration. Moreover, **1** and **2** showed almost the same CD curve (Figure 5), further confirming their same absolute configuration.

Tanzawaic acids T (**3**) and U (**4**) were obtained as pale yellow oil, and had the same molecular formula (C_19_H_28_O_3_), established based on the same HRESIMS ions both detected at *m*/*z* 303.1962 [M − H]^−^. Their 1D NMR data (Table 2) suggested that they possessed similar tanzawaic acid scaffold to Compound **2**. Further analysis of the 2D NMR spectra indicated that Compounds **3** and **4** shared the same planar structure (Figure 2), and the structural difference between **3** (or **4**) and **2** was that the hydroxymethyl in **2** was replaced by a methyl in **3** and **4** (δ_H_ 0.89 in **3**, δ_H_ 0.88 in **4**). The NOESY spectroscopic data of **3** and **4** indicated that they are C-13 epimers (Figure 3). The NOESY correlations between H-7 and H-13 suggested the β orientation of H-13 in Compound **3**. The NOESY correlations between H-13 and H-11a (δ_H_ 1.57), H-11a and H-10, and between H-11b (δ_H_ 1.16) and H_3_-17/13-OCH_3_ in Compound **4** indicated an *α* oriented H-13 in **4** (Figure 3). Although the relative configuration of C-13 in Compound **3** was different from **1**, the CD curves of them are almost identical (Figure 5), indicating that the cotton effects, especially those at 270 nm, were predominated by the (2*E*, 4*E*)-penta-2,4-dienoic acid moiety. Consequently, the absolute configurations of **3** and **4** were deduced as 6*R*, 7*R*, 8*R*, 10*S*, 12*S*, 13*R* and 6*R*, 7*R*, 8*R*, 10*S*, 12*S*, 13*S*, respectively.

Tanzawaic acid V (**5**) was isolated as a pale yellow oil with the molecular formula of C_18_H_26_O_4_ based on the HRESIMS ions detected at *m*/*z* 305.1751 [M − H]^−^. The 1D NMR data (Table 3) indicated the presence of three methyls, two methylene, ten methines (including six sp^2^ methines), and three non-protonated carbons (including one carbonyl and two oxygenated carbons). These data are similar to those of the isolated known compound, tanzawaic acid C (**7**) [19], except for the appearance of an additional oxygenated non-protonated carbon and the absence of a methine in **5**, indicating that Compound **5** is a hydroxylated analogue of **7**. Analysis of the 2D NMR spectra located the additional hydroxyl group on C-10 (Figure 2), and the methyl group on C-10 in **5** oriented to the same side as in Compound **7** according to the NOESY correlation between H_3_-17 and H_3_-18 (Figure 3). The absolute configuration of **5** was determined by comparing CD with that of **7** whose absolute configuration was determined using X-ray diffraction with Flack parameter = 0.06 (9) in this work (Figure 4). Their identical CD curve indicated the same absolute configuration (Figure 5).

Tanzawaic acid W (**6**) was isolated as a pale yellow oil, whose molecular formula was established as identical to that of Compound **5** based on the HRESIMS ions detected at *m*/*z* 305.1752 [M − H]^−^. The 1D and 2D NMR spectra indicated that the difference to **5** was the lack of the hydroxyl group at C-10 and its presence at C-15, and the methyl on C-15 faced to the same orientation to H-7 according to the NOESY correlations between H-7 and H_3_-16. Compound **6** also had similar CD curve with **7**, which indicated the same absolute configuration (Figure 5).

Tanzawaic acid X (**11**) was isolated as pale yellow oil with the molecular formula of C_18_H_22_O_3_ based on the HRESIMS ions detected at *m*/*z* 285.1493 [M − H]^−^. The 1D NMR data (Table 3) indicated the presence of two methyls, three methylenes (with one oxygenated), eight methines (including six sp^2^ ones), and five non-protonated carbons (including one carbonyl). Further analysis of the 2D NMR spectra indicated that the planar structure **11** was similar to tanzawaic acid A (**13**) [19] and the only difference was the methyl at C-10 in **13** was replaced by a hydroxymethyl. The relative configuration of **11** was determined by NOESY correlations between H-8 (δ_H_ 3.26) and H-10 (δ_H_ 1.85), which suggested the cofacial of H-8 and H-10. Moreover, the coincident CD curves of **11** and **13** indicated the 8*R*, 9*S* absolute configuration of **11** (Figure 5).

The seven known Compounds **7**–**10** and **12**–**14** were identified (Figure 1) as tanzawaic acid C (**7**), tanzawaic acid B (**8**), tanzawaic acid M (**9**), tanzawaic acid E (**10**), arohynapene B (**12**), tanzawaic acid A (**13**) and tanzawaic acid D (**14**) by comparing NMR data and optical rotation with those reported in the literature [15,19,20,21,22,26]. As determining the absolute configuration of tanzawaic acid derivatives always be a challenging work, the stereochemistry of Compounds **7**–**9**, **12** and **14** were not confirmed by solid evidence in literature reports. Herein, we present the determination of the absolute configurations assisted by X-ray differentiation, CD data and chemical transformation. The absolute configurations of Compounds **7** and **8** were determined under X-ray (CCDC numbers 1537544 and 1537542) with the Flack parameters = 0.06 (9) and 0.07 (10), respectively (Figure 4). The absolute configurations of Compounds **9** and **14** were determined by comparing CD spectra with Compounds **8** and **13**, respectively (Figure 5). The absolute configuration of Compound **12** was assigned by chemical transformation. We found the Pd/C reduction products (established as **12a**) of Compounds **12** and **13** showed the same 1D NMR data and similar optical rotations (${\left[\alpha \right]}_{D}^{20}$−7.78 (c 0.042, MeOH) and ${\left[\alpha \right]}_{D}^{20}$ −6.09 (c 0.042, MeOH), respectively), which suggested Compounds **12** and **13** had the same relative and absolute configurations, which is also in accord with the same CD curves of **12** and **13** (Figure 5).

**Figure 6:**
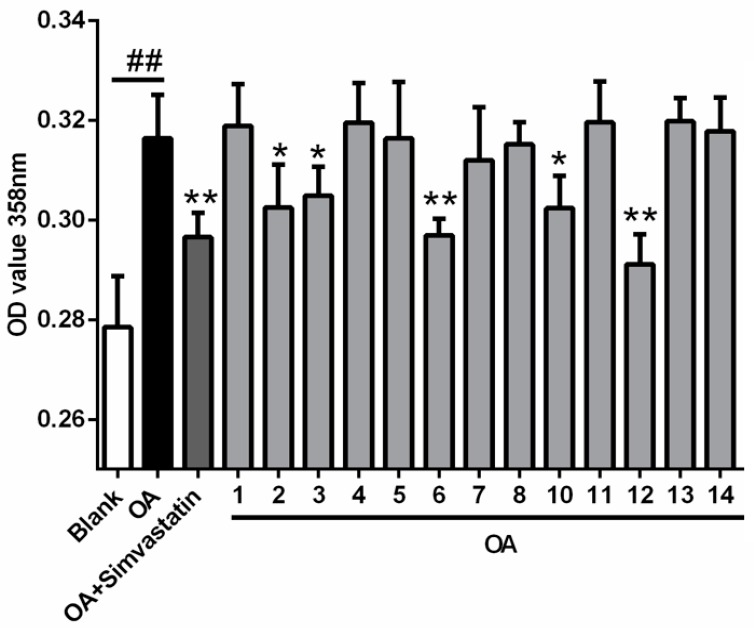
Effects of compounds on OA-elicited intracellular lipid accumulation. Cells were treated with 10 μM of indicated compounds or simvastatin (as positive control) in DMEM + 100 μM OA or with DMEM alone (as blank) or DMEM + 100 μM OA (as negative control) for 24 h Neutral lipids were determined by spectrophotometry at 358 nm after oil-red O staining. Bars depict the means ± SEM of at least three experiments. ^##^ *p* < 0.01, OA versus Blank; * *p* < 0.05, ** *p* < 0.01, test group versus OA group. OA: oleic acid.

**Figure 7:**
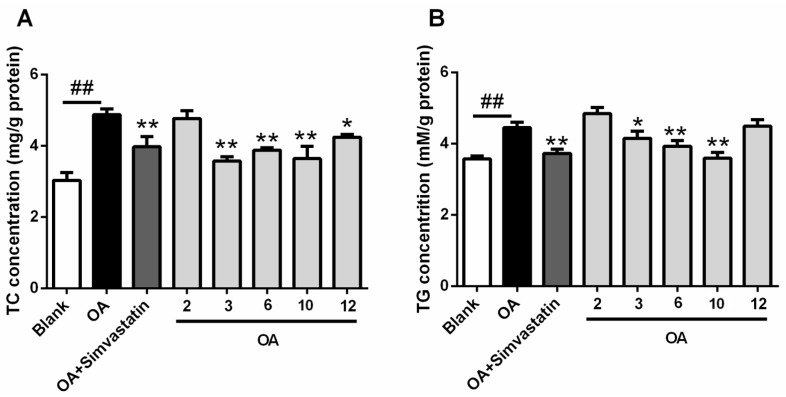
Inhibitory effects of compounds on intracellular (**A**) total cholesterol (TC) and (**B**) triglycerides (TG). Cells were treated with 10 μM of indicated compounds or simvastatin (as positive control) in DMEM + 100 μM OA or with DMEM alone (as blank) or DMEM + 100 μM OA (as negative control) for 24 h. Intracellular TC and TG concentration were measured by kits according to the manufacturer’s instructions. Bars depict the means ± SEM of at least three experiments. ^##^ *p* < 0.01, OA versus blank; * *p* < 0.05, ** *p* < 0.01, test group versus OA group. OA: oleic acid.

All the compounds were evaluated for their cytotoxicity (on HL-60, HCT-116, K562, Hela and A549 cell lines), but none of them presented a cytotoxic effect at 30 μM. The antiviral (influenza A H1N1 virus) and NF-κB inhibitory activities were also evaluated, with no activity detected under the concentration of 30 μM. In light of the structural similarity with lovastatin, with the exception of Compound **9** (limited quantity), all the compounds were evaluated for their lowering effects against oleic acid (OA)-elicited lipid accumulation in HepG2 liver cells. Five compounds (**2**, **3**, **6**, **10** and **12**) significantly decreased the lipid accumulation elicited by OA, determined by oil-red O staining, at the concentration of 10 μM. Compounds **6** and **12** showed comparable efficiency with simvastatin (Figure 6). Additionally, four compounds (**3**, **6**, **10** and **12**) could significantly decreased intracellular total cholesterol (TC) levels and three compounds (**3**, **6**, and **10**) significantly decreased intracellular triglyceride (TG) levels (Figure 7). It’s worth mentioning that the TG-lowering efficiency of Compounds **6** and **10** were comparable with simvastatin and the TG levels were nearly equal to blank control (*p* > 0.05) (Figure 7B).

## 3. Materials and Methods

### 3.1. General Experimental Procedures

UV spectra were recorded on Beckman DU 640 spectrophotometer (Beckman Coulter Inc., Brea, CA, USA). IR spectra were taken on Bruker tensor-27 spectrophotometer in KBr discs (Bruker Corporation, Billerica, MA, USA). Specific rotations were measured on JASCO P-1020 digital polarimeter (JASCO Corporation, Tokyo, Japan). ESIMS were obtained on Thermo Scientific LTQ Orbitrap XL mass spectrometer (Thermo Fisher Scientific, Waltham, MA, USA) or Micromass Q-TOF ULTIMA GLOBAL GAA076 LC Mass spectrometer (Wasters Corporation, Milford, MA, USA). CD spectra were measured on JASCO J-715 spectropolarimeter (JASCO Corporation, Tokyo, Japan). NMR spectra were recorded on Agilent 500 MHz DD2 spectrometer using TMS as internal standard and chemical shifts were recorded as δ-values (Agilent Technologies Inc., Santa Clara, CA, USA). Semi-preparative HPLC was performed on an ODS column (HPLC (YMC-Pack ODS-A, 10 × 250 mm, 5 μm, 3 mL/min)) (YMC Co., Ltd., Kyoto, Japan). Medium-pressure preparation liquid chromatography (MPLC) was performed on a Bona-Agela CHEETAHTM HP100 (Beijing Agela Technologies Co., Ltd., Beijing, China). Column chromatography (CC) were performed with silica gel (200–300 mesh, Qingdao Marine Chemical Inc., Qingdao, China), and Sephadex LH-20 (Amersham Biosciences, San Francisco, CA, USA), respectively [27].

### 3.2. Fungal Material

The fungal strain *P. steckii* HDN13-279 was isolated from the leaf of *Sonneratia caseolaris* collected from mangrove conservation area of Hainan, China. It was identified by ITS sequence and the sequence data have been submitted to GenBank (accession number: KY399997). The voucher specimen was deposited in our laboratory at −20 °C.

### 3.3. Fermentation and Extraction

The fungus *P. steckii* HDN13-279 was cultured at 28 °C on a rotary platform shakers at 180 rpm for 9 days in 500 mL Erlenmeyer flasks containing 150 mL of liquid culture medium, composed of maltose (20.0 g/L), mannitol (20.0 g/L), glucose (10.0 g/L), monosodium glutamate (10.0 g/L), MgSO_4_·7H_2_O (0.3 g/L), KH_2_PO_4_ (0.5 g/L), yeast extract (3.0 g/L), corn steep liquor (1.0 g/L), and seawater (Huiquan Bay, Yellow Sea, Qingdao, China). After 9 days of cultivation, 45 L of whole broth was filtered through cheesecloth to separate the supernatant from the mycelia. The supernatant was extracted three times with EtOAc. The water-containing mycelia was extracted three times with acetone, and the mixed solution was concentrated under reduced pressure to afford an aqueous solution without acetone, then extracted three times with EtOAc. Both EtOAc solutions were combined and concentrated under reduced pressure to give the organic extract (40 g) [28].

### 3.4. Isolation

The organic extract was subjected to vacuum liquid chromatography over a silica gel column using a gradient elution with petroleum ether-CHCl_3_-MeOH to give six fractions (fractions 1–6). Fraction 3 (5.1 g) eluted with 98:2 CH_2_Cl_2_-MeOH was applied on a C-18 ODS column using a stepped gradient elution of MeOH-H_2_O yielding five subfractions (fractions 3.1–3.5). Fraction 3.3 that eluted with 50:50 MeOH-H_2_O was separated by semi-preparative HPLC using a stepped gradient elution of CH_3_CN-H_2_O (25:75 to 65:35, 3 mL/min) to furnish Compounds **1** (10.0 mg, *t*_R_ 14.5 min), **11** (7.0 mg, *t*_R_ 16.7 min), **5** (4.0 mg, *t*_R_ 19.2 min), **10** (5.0 mg, *t*_R_ 23.8 min), **2** (4.0 mg, *t*_R_ 25.5 min), and **9** (4.0 mg, *t*_R_ 28.2 min). Fraction 3.4 that eluted with 75:25 MeOH-H_2_O was chromatographed on Sephadex LH-20 CH_2_Cl_2_-MeOH (1:1) and further separated by MPLC (C-18 ODS) using MeOH-H_2_O (70:30) to furnish four subfractions (fractions 3.4.1–3.4.4). Fraction 3.4.1 was purified by semi-preparative HPLC (63:37 MeCN:H_2_O, 3 mL/min) to afford Compounds **3** (14.0 mg, *t*_R_ 20.5 min) and **4** (15.0 mg, *t*_R_ 24.2 min). Fraction 3.4.2 was purified by semi-preparative HPLC (73:27 MeOH:H_2_O, 3 mL/min) to afford Compounds **14** (8.0 mg, *t*_R_ 15.4 min), **6** (5.0 mg, *t*_R_ 21.4 min), **12** (10.0 mg, *t*_R_ 24.4 min), and **7** (18.0 mg, *t*_R_ 27.5 min). Fraction 3.4.4 was purified by semi-preparative HPLC (80:20 MeOH:H_2_O, 3 mL/min) to afford Compounds **13** (20.0 mg , *t*_R_ 16.4 min) and **8** (25.0 mg, *t*_R_ 20.4 min).

*Tanzawaic acid R* (**1**): Pale yellow oil; ${\left[\alpha \right]}_{D}^{20}$ +49.4 (*c* 0.57, MeOH); CD (1.63 × 10^−3^ M, MeOH) λ [nm] (Δε): 263 (76.2), 219 (−2.9); IR (KBr) *ν*_max_ 3415, 2921, 1685, 1413, 1207, 1005, 724 cm^−1^; UV (MeOH) *λ*_max_ (log ε): 221 (1.10), 268 (4.06) nm; ^1^H and ^13^C NMR data, see Table 1; HRESIMS [M − H]^−^ *m*/*z* 305.1750 (calcd. for C_18_H_25_O_4_, 305.1747).

*Tanzawaic acid S* (**2**): pale yellow oil; ${\left[\alpha \right]}_{D}^{20}$ +126.4 (*c* 0.25, MeOH); CD (1.56 × 10^−3^ M, MeOH) *λ* [nm] (Δε): 263 (85.8), 220 (−10.1); IR (KBr) *ν*_max_ 3414, 2927, 1684, 1412, 1006, 622 cm^−1^; UV (MeOH) *λ*_max_ (log ε): 221 (1.04), 268 (4.01) nm; ^1^H and ^13^C NMR data, see Table 1; HRESIMS [M − H]^−^ *m*/*z* 319.1906 (calcd. C_19_H_27_O_4_, 319.1915).

*Tanzawaic acid T* (**3**): pale yellow oil; ${\left[\alpha \right]}_{D}^{20}$ +98.2 (*c* 0.27, MeOH); CD (1.64 × 10^−3^ M, MeOH) *λ* [nm] (Δε): 266 (102.4), 216 (−5.6); IR (KBr) *ν*_max_ 3416, 2910, 1688, 1453, 1272, 1092, 1003, 562 cm^−1^; UV (MeOH) *λ*_max_ (log ε): 221 (1.05), 261 (3.99) nm; ^1^H and ^13^C NMR data, see Table 2; HRESIMS [M − H]^−^ *m*/*z* 303.1962 (calcd. C_19_H_27_O_3_, 303.1955).

*Tanzawaic acid U* (**4**): pale yellow oil; ${\left[\alpha \right]}_{D}^{20}$ +66.9 (*c* 0.20, MeOH); CD (1.64 × 10^−3^ M, MeOH) *λ* [nm] (Δε): 266 (96.0), 220 (−10.1); IR (KBr) *ν*_max_ 2949, 2910, 1688, 1417, 1272, 1092, 1003 cm^−1^; UV (MeOH) *λ*_max_ (log ε): 221 (1.02), 261 (3.96) nm; ^1^H and ^13^C NMR data, see Table 2; HRESIMS [M − H]^−^ *m*/*z* 303.1962 (calcd. C_19_H_27_O_3_, 303.1955).

*Tanzawaic acid V* (**5**): pale yellow oil; ${\left[\alpha \right]}_{D}^{20}$ +18.9 (*c* 0.30, MeOH); CD (1.63 × 10^−3^ M, MeOH) *λ* [nm] (Δε): 275 (4.9), 253 (−2.2), 224 (1.4); IR (KBr) *ν*_max_ 3396, 2929, 1689, 1377, 1152, 1011, 732 cm^−1^; UV (MeOH) *λ*_max_ (log ε): 221 (2.30), 239 (3.56) nm, 284 (4.06) nm; ^1^H and ^13^C NMR data, see Table 3; HRESIMS [M − H]^−^ *m*/*z* 305.1751 (calcd. for C_18_H_25_O_4_, 305.1758).

*Tanzawaic acid W* (**6**): pale yellow oil; ${\left[\alpha \right]}_{D}^{20}\;$ −5.9 (*c* 0.17, MeOH); CD (1.63 × 10^−3^ M, MeOH) *λ* [nm] (Δε): 298 (2.6), 258 (−9.6), 227 (1.5); IR (KBr) *ν*_max_ 3416, 2924, 1702, 1459, 1377, 1261, 1013, 748 cm^−1^; UV (MeOH) *λ*_max_ (log ε): 221 (1.20), 264 (4.09) nm; ^1^H and ^13^C NMR data, see Table 3; HRESIMS [M − H]^−^ *m*/*z* 305.1752 (calcd. for C_18_H_25_O_4_, 305.1747).

*Tanzawaic acid C* (**7**): CD (1.72 × 10^−3^ M, MeOH) *λ* [nm] (Δε): 251 (−1.7), 227 (0.5), 220 (−3.2).

*Tanzawaic acid B* (**8**): CD (1.82 × 10^−3^ M, MeOH) *λ* [nm] (Δε): 270 (12.7), 220 (−3.2).

*Tanzawaic acid M* (**9**): CD (1.72 × 10^−3^ M, MeOH) *λ* [nm] (Δε): 269 (8.0), 220 (−3.7).

*Tanzawaic acid X* (**11**): pale yellow oil; ${\left[\alpha \right]}_{D}^{20}$ +131.1 (*c* 0.20, MeOH); CD (1.75 × 10^−3^ M, MeOH) *λ* [nm] (Δε): 294 (28.5), 238 (−4.0), 218 (3.3); IR (KBr) *ν*_max_ 3392, 2925, 1695, 1390, 1273, 996, 871, 808 cm^−1^; UV (MeOH) *λ*_max_ (log ε): 221 (2.32), 239 (3.50) nm, 293 (4.09) nm; ^1^H and ^13^C NMR data, see Table 3; HRESIMS [M − H]^−^ *m*/*z* 285.1493 (calcd. for C_18_H_21_O_3_, 285.1485).

*Arohynapene B* (**12**): CD (1.75 × 10^−3^ M, MeOH) *λ* [nm] (Δε): 292 (26.5), 235 (−3.6), 217 (1.6).

*Tanzawaic acid A* (**13**): CD (1.85 × 10^−3^ M, MeOH) *λ* [nm] (Δε): 295 (31.5), 236 (−4.6), 217 (0.1).

*Tanzawaic acid D* (**14**): CD (1.75 × 10^−3^ M, MeOH) *λ* [nm] (Δε): 291 (33.7), 237 (−3.7), 218 (7.1).

### 3.5. Crystal Data for ***1a***

Orthorhombic, C_19_H_28_O_4_, space group P2_1_2_1_2_1_, *a* = 7.69910 (10) Å, *b* = 12.8933 (2) Å, *c* = 18.6839 (3) Å, *V* = 1854.69 (5) Å^3^, *Z* = 4, *T* = 290 (2) K, μ (CuKα) = 0.635 mm^−1^, *D*_calcd._ = 1.147 g/cm^3^, 13,363 reflections measured (8.332° ≤ 2*θ* ≤ 139.834°), 3474 unique (*R*_int_ = 0.0298, *R*_sigma_ = 0.0268) which were used in all calculations. The final *R*_1_ was 0.0383 and *wR*_2_ was 0.0955 (*I* > 2*σ*(*I*)). Flack parameter = 0.02 (11).

### 3.6. Crystal Data for ***7***

Orthorhombic, C_18_H_26_O_3_, space group P2_1_2_1_2_1_, *a* = 7.1226 (2) Å, *b* = 12.8012 (3) Å, *c* = 37.9453 (12) Å, *V* = 3459.77 (17) Å^3^, *Z* = 8, *T* = 291 (2) K, μ (CuKα) = 0.589 mm^−1^, *D*_calcd._ = 1.115 g/cm^3^, 30,947 reflections measured (7.288° ≤ 2*θ* ≤ 142.662°), 6596 unique (*R*_int_ = 0.0332, *R*_sigma_ = 0.0254) which were used in all calculations. The final *R*_1_ was 0.0408 and *wR*_2_ was 0.0982 (*I* > 2*σ*(*I*)). Flack parameter = 0.06 (9).

### 3.7. Crystal Data for ***8***

Orthorhombic, C_18_H_26_O_2_, space group P2_1_2_1_2_1_, *a* = 7.2748 (2) Å, *b* = 12.7225 (3) Å, *c* = 36.8100 (12) Å, *V* = 3406.90 (17) Å^3^, *Z* = 8, *T* = 289 (2) K, μ (CuKα) = 0.527 mm^−1^, *D*_calcd._ = 1.070 g/cm^3^, 22,920 reflections measured (7.352° ≤ 2*θ* ≤ 140.302°), 6368 unique (*R*_int_ = 0.0271, *R*_sigma_ = 0.0284) which were used in all calculations. The final *R*_1_ was 0.0448 and *wR*_2_ was 0.1130 (*I* > 2*σ*(*I*)). Flack parameter = 0.07 (10).

### 3.8. X-ray Crystallographic Analysis of Compound ***1a***, ***7*** and ***8***

Crystals of **1a**, **7** and **8** were obtained in the mixed solvent of CHCl_3_-MeOH, and crystallographic data for **1a**, **7** and **8** (Cu Kα radiation) have been deposited in the Cambridge Crystallographic Data Center with the deposition numbers CCDC 1537543, 1537544 and 1537542, respectively. These data can be obtained free of charge from the Cambridge Crystallographic Data Centre via the link of reference [29].

### 3.9. Esterification of ***1***

To a solution of **1** (3.8 mg) in MeOH (0.5 mL) was added excess TMS-CHN_2_ in *n*-hexane (300 µL), and the mixture was stirred at r. t. for 30 min. Then the reaction mixture was concentrated in vacuo and the residue was purified by preparative HPLC (MeOH: H_2_O = 50–100%) to yield methyl ester **1a** (2.0 mg). **1a**: ^1^H NMR (500 MHz, Methanol-*d*_4_) δ 7.26 (1H, dd, *J* = 11.1, 15.4 Hz, H-3), 6.33 (1H, dd, *J* = 11.0, 15.3 Hz, H-4), 6.05 (1H, dd, *J* = 9.5, 15.3 Hz, H-5), 5.85 (1H, d, *J* = 15.4 Hz, H-2), 5.82 (1H, d, *J* = 6.5 Hz, H-14), 3.82 (1H, dd, *J* = 1.7, 6.5 Hz, H-13), 3.71 (3H, s, -CH_3_), 3.36 (2H, d, *J* = 6.3 Hz, H_2_-17), 2.61 (1H, t, *J* = 7.9 Hz, H-6), 1.68 (1H, overlap, H-11a), 1.66 (1H, overlap, H-9a), 1.60 (3H, s, H_3_-16), 1.60 (1H, overlap, H-10), 1.36 (1H, overlap, H-8), 1.33 (1H, overlap, H-7), 1.28 (1H, overlap, H-12), 1.12 (1H, q, *J* = 12.1 Hz, H-11b), 0.96 (3H, d, *J* = 6.3 Hz, H_3_-18), 0.73 (1H, q, *J* = 12.0 Hz, H-9b).

### 3.10. Methylation of ***1*** and ***2***

To a solution of **1** (1.6 mg) or **2** (1.6 mg) and excess NaH in DMF (0.2 mL) were added excess CH_3_I, and the mixtures were stirred at 80 °C for 6 h. Then the reaction mixtures were filtered and purified by preparative HPLC (MeOH:H_2_O = 50–100%) to yield **1b** (0.8 mg from **1** and 0.7 from **2**), respectively. **1b**: ^1^H NMR (500 MHz, Methanol-*d*_4_) δ 7.24 (1H, dd, *J* = 11.6, 15.3 Hz, H- 3), 6.30 (1H, dd, *J* = 11.0, 15.3 Hz, H-4), 5.96 (1H, overlap, H-14), 5.96 (1H, overlap, H-5), 5.81 (1H, d, *J* = 15.4 Hz, H-2), 3.45 (1H, d, *J* = 5.9 Hz, H-13), 3.33 (3H, s, OCH_3_-13), 3.31 (3H, s, OCH_3_-17), 3.21 (2H, d, *J* = 6.3 Hz, H_2_-17), 2.61 (1H, dd, *J* = 4.6, 8.8 Hz, H-6), 1.70 (1H, overlap, H-11a), 1.68 (1H, overlap, H-9a), 1.64 (3H, s, H_3_-16), 1.61 (1H, overlap, H-10), 1.31 (1H, overlap, H-8), 1.31 (1H, overlap, H-7), 1.31 (1H, overlap, H-12), 1.19 (1H, q, *J* = 11.6 Hz, H-11b), 0.95 (3H, d, *J* = 5.7 Hz, H_3_-18), 0.75 (1H, q, *J* = 10.8 Hz, H-9b). ^13^C NMR (125 MHz, Methanol-*d*_4_) δ 174.7, 144.9, 140.4, 139.6, 130.4, 127.0, 123.1, 78.3, 76.3, 57.6, 55.3, 50.0, 44.5, 42.5, 39.7, 39.7, 37.5, 33.0, 21.2, 21.1. ESI-MS [M − H]^−^ *m*/*z* 333.45.

### 3.11. Reduction of ***12*** and ***13***

To a solution of **12** (2.0 mg) or **13** (2.0 mg) in MeOH (300 µL) were added Pd/C (0.2 mg) under hydrogen atmosphere. After stirring at r.t. for 2 h, the reaction mixtures were evaporated in vacuo, and **12a** (0.6 mg (${\left[\alpha \right]}_{D}^{20}$ −7.78 (*c* 0.042, MeOH)) from **12** and 0.6 mg (${\left[\alpha \right]}_{D}^{20}$ −6.09 (*c* 0.042, MeOH)) from **13**) were obtained by semi-preparative HPLC using a gradient solvent system of 30−100% CH_3_CN/H_2_O over 30 min, respectively. **12a**: ^1^H NMR (500 MHz, CDCl_3_) δ 6.90 (1H, d, *J* = 7.6 Hz), 6.83 (1H, d, *J* = 7.6 Hz), 3.22 (1H, m), 2.72–2.59 (2H, overlap), 2.58 (1H, m), 2.41 (2H, t, *J* = 7.5 Hz), 2.36 (1H, dd, *J* = 12.4, 14.1 Hz), 2.29 (3H, s), 2.19 (1H, m), 1.77 (2H, m), 1.63 (1H, m), 1.51 (2H, m), 1.18 (3H, d, *J* = 7.1 Hz), 1.14 (1H, m), 1.07 (1H, d, *J* = 6.6 Hz). ^13^C NMR (125 MHz, CDCl_3_) δ 178.7, 140.4, 137.9, 136.9, 134.0, 127.5, 126.1, 40.1, 39.0, 33.7, 30.0, 29.9, 29.8, 28.6, 25.6, 25.3, 22.9, 19.8.

### 3.12. Cell-Based Lipid Accumulation Assay

HepG2 cells, seeded in a 96 wells plate at the concentration of 1 × 10^5^ cells/well, were cultured in high glucose DMEM medium containing 10% fetal bovine serum (FBS) at 37 °C and 5% CO_2_. After reaching 90% confluence, cells were incubated with the indicated concentration of compounds (10 µM) or with simvastatin (10 μM) in high glucose DMEM containing OA (100 μM) for 24 h. The blank group was incubated with serum-free high glucose DMEM alone. Oil red O staining was performed as previous reported [30] and the intracellular contents of total cholesterol and triglyceride were determined by kits according to manufacturer’s instructions.

### 3.13. Assay of Cytotoxicity, Antiviral Activity and NF-κB Inhibitory Activity

These biological evaluations were carried out as previously reported [27,31,32].

### 3.14. Statistical Analysis

The data of lipid lowering effect were expressed as mean ± SEM, representing at least three different experiments with *n* = 8 in each test. SPSS 17.0 software (SPSS, Chicago, IL, USA) was used for statistical analysis. Differences were assessed by an unpaired *t*-test. A probability level (*p*) of 0.05 was considered significant.

## 4. Conclusions

In summary, seven new tanzawaic acid derivatives, along with seven known compounds were isolated from *Penicillium steckii* HDN13-279. The absolute configurations of all the compounds (including the known compounds of which the absolute configurations were not confirmed in the literature) were determined by NMR, X-ray diffraction, CD analyses, as well as chemical derivatization. In addition, for the first time, we evaluated the lowering effects against oleic acid (OA)-elicited lipid accumulation in HepG2 liver cells of this kinds of compounds, and five (**2**, **3**, **6**, **10** and **12**) showed pharmaceutical potential with lipid-lowering activity.

## Figures and Tables

**Table 1:** ^1^H (500 MHz) and ^13^C (125 MHz) NMR Data of Compounds **1** and **2** (Methanol-*d*_4_, δ ppm).

No.	1		2	
	δ_C_	δ_H_ (*J* in Hz)	δ_C_	δ_H_ (*J* in Hz)
1	170.1	-	169.3	-
2	120.3	5.82 overlap	119.4	5.80 d (15.3)
3	144.6	7.22 dd (11.1, 15.3)	145.1	7.23 dd (11.0, 15.3)
4	129.8	6.32 dd (10.6, 15.3)	129.4	6.29 dd (11.0, 15.3)
5	149.1	6.00 dd (9.4, 15.3)	149.3	5.96 overlap
6	50.3	2.61 t (8.0)	50.1	2.61 dd (4.7, 9.0)
7	43.7	1.33 m	44.5	1.32 overlap
8	39.6	1.37 m	39.7	1.32 overlap
9	39.6	1.69 overlap	39.5	1.69 overlap
		0.74 q (12.2)		0.72 q (11.8)
10	39.9	1.61 overlap	39.9	1.57 m
11	32.6	1.66 overlap	32.7	1.68 overlap
		1.13 q (12.1)		1.17 q (12.2)
12	42.6	1.29 m	42.4	1.32 overlap
13	67.0	3.83 dd (2.3, 6.4)	76.3	3.46 d (6.1)
14	125.5	5.83 overlap	123.7	5.96 overlap
15	138.2	-	139.7	-
16	21.1	1.61 s	21.1	1.63 s
17	67.3	3.37 d (6.2)	67.3	3.35 d (6.6)
18	21.5	0.97 d (6.1)	21.1	0.95 d (5.8)
13-OCH_3_	-	-	55.5	3.33 s

**Table 2:** ^1^H (500 MHz) and ^13^C (125 MHz) NMR Data of Compounds **3** and **4** (CDCl_3_, δ ppm).

No.	3		4	
	δ_C_	δ_H_ (*J* in Hz)	δ_C_	δ_H_ (*J* in Hz)
1	172.0	-	172.3	-
2	118.5	5.80 d (15.4)	118.5	5.79 d (15.3)
3	147.0	7.33 dd (10.9, 15.1)	147.2	7.33 dd (11.0, 15.3)
4	129.0	6.19 dd (11.0, 15.2)	129.0	6.21 dd (11.2, 15.3)
5	150.7	5.92 dd (9.4, 15.2)	151.0	6.04 dd (9.4, 15.3)
6	49.9	2.57 t (8.4)	50.3	2.53 d (5.9, 8.1)
7	48.7	0.94 m	44.1	1.30 overlap
8	39.7	1.38 m	40.1	1.30 overlap
9	45.7	1.62 overlap	45.2	1.55 overlap
		0.75 q (12.3)		0.77 q (11.9)
10	31.5	1.44 m	32.3	1.46 m
11	38.2	2.19 d (3.2, 12.6)	38.5	1.57 overlap
		0.61 q (12.2)		1.16 q (12.0)
12	43.9	1.23 m	42.8	1.30 overlap
13	80.6	3.32 d (10.2)	76.4	3.41 d (6.2)
14	126.0	5.66 s	124.3	5.92 d (5.9)
15	134.7	-	139.5	-
16	21.8	1.58 s	22.5	1.61 s
17	22.5	0.89 overlap	22.4	0.88 overlap
18	22.4	0.89 overlap	21.8	0.88 overlap
13-OCH_3_	56.0	3.37 s	56.7	3.35 s

**Table 3:** ^1^H (500 MHz) and ^13^C (125 MHz) NMR Data of Compounds **5**, **6** and **11** (δ ppm).

No.	5 *^a^*		6 *^b^*		11 *^b^*	
	δ_C_	δ_H_ (*J* in Hz)	δ_C_	δ_H_ (*J* in Hz)	δ_C_	δ_H_ (*J* in Hz)
1	169.8	-	171.5	-	171.6	-
2	120.0	5.84 d (15.3)	120.0	5.90 d (15.6)	119.8	5.94 d (15.3)
3	144.9	7.34 dd (10.0, 15.4)	145.9	7.42 dd (11.6, 14.4)	147.1	7.58 dd (11.0, 15.2)
4	122.3	6.47 dd (9.9, 15.4)	125.4	6.51 dd (11.5, 14.4)	131.2	6.43 dd (11.0, 15.8)
5	153.5	6.52 d (15.3)	149.0	6.40 d (15.2)	141.4	7.12 d (15.9)
6	75.8	-	77.9	-	135.0	-
7	49.1	1.18 overlap	51.9	1.35 t (9.8)	140.8	-
8	29.0	2.00 m	33.7	1.69 m	30.4	3.26 m
9	50.1	1.62 dt (3.2, 14.1)	47.1	1.66 overlap	35.0	2.18 m
		1.23 overlap		0.81 overlap		1.19 m
10	69.1	-	32.6	1.56 m	37.3	1.85 m
11	45.3	1.72 dt (3.0, 13.4)	41.4	1.78 d (12.2)	33.6	2.77 dt (3.05,14.5)
		1.25 m		0.83 overlap		2.46 dd (11.9, 15.1)
12	33.4	2.58 m	38.3	2.21 t (10.7)	135.2	-
13	130.1	5.36 d (9.8)	132.5	5.49 d (9.9)	128.7	6.98 overlap
14	129.1	5.50 m	130.1	5.40 d (9.9)	127.8	6.98 overlap
15	45.7	-	75.0	-	134.1	-
16	18.3	0.97 d (7.1)	27.4	1.27 s	21.1	2.28 s
17	30.1	1.18 s	22.2	0.88 d (6.5)	68.0	3.66 overlap
						3.65 overlap
18	22.0	0.91 d (6.6)	23.5	0.95 d (6.1)	24.0	1.16 d (7.0)

*^a^* The NMR data were recorded in Methanol-*d*_4_; *^b^* the NMR data were recorded in CDCl_3_.

## Supplementary Materials

The following are available online at www.mdpi.com/1660-3397/16/1/25/s1, 1D and 2D NMR spectra, IR spectra of **1**–**6**, **11**; ^1^H NMR spectrum of **1a**; 1D NMR spectra of **1b** and **12a**. Figures S1–S7: 1D, 2D NMR, NOESY and HRESIMS spectra of tanzawaic acid R (**1**), Figures S8–S14: 1D, 2D NMR, NOESY and HRESIMS spectra of tanzawaic acid S (**2**), Figures S15–S21: 1D, 2D NMR, NOESY and HRESIMS spectra of tanzawaic acid T (**3**), Figures S22–S28: 1D, 2D NMR, NOESY and HRESIMS spectra of tanzawaic acid U (**4**), Figures S29–S35: 1D, 2D NMR, NOESY and HRESIMS spectra of tanzawaic acid V (**5**), Figures S36–S42: 1D, 2D NMR, NOESY and HRESIMS spectra of tanzawaic acid W (**6**), Figures S43–S49: 1D, 2D NMR, NOESY and HRESIMS spectra of tanzawaic acid X (**11**), Figure S50: ^1^H NMR spectrum of **1a**, Figures S51–S52: 1D NMR spectra of **1b**, Figures S53–S54: 1D NMR spectra of **12a**, Figures S55–S61: IR spectrum of tanzawaic acids R–X (**1**–**6**, **11**).
