# Supplementary material for: Lipid-Lowering Polyketides from the Fungus Penicillium Steckii HDN13-279
Source: Mar Drugs. 2018 Jan 12;16(1):25. doi: 10.3390/md16010025 (PMC5793073; doi:10.3390/md16010025)

Supporting Information

Lipid-lowering Polyketides from the Fungus *Penicillium steckii* HDN13-279

Gui-Hong Yu, [1,†] Shuai Wang, [2,†] Lu Wang, [1] Qian Che, [1] Tian-Jiao, Zhu, [1] Guo-Jian Zhang, [1,3] Qian-Qun Gu, [1] Peng Guo [2,*] and De-Hai Li [1,3,*]

[1] Key Laboratory of Marine Drugs, Chinese Ministry of Education, School of Medicine and Pharmacy, Ocean University of China, Qingdao 266003, P. R. China; Yuguihong1990@126.com (G.-H. Y.); 784940819@qq.com (L. W.); cheqian064@ouc.edu.cn (Q. C.); zhutj@ouc.edu.cn (T.-J. Z); guqianq@ouc.edu.cn (Q.-Q. G.)

[2] Institute of Medicinal Plant Development, Chinese Academy of Medical Sciences & Peking Union Medical College, Beijing 100193, P. R. China; zhuizhirun@163.com (S. W.)

[3] Laboratory for Marine Drugs and Bioproducts of Qingdao National Laboratory for Marine Science and Technology, Qingdao, 266237, P. R. China; 95327787@qq.com (G.-J. Z.)

***** Correspondence: E-mail: dehaili@ouc.edu.cn (D.-H. L.) and pguo@implad.ac.cn (P. G.). Tel.: 0086-532-82032066 (D.-H. L.).

[†] G.-H. Y. and S. W. contributed equally.

List of Supporting Information

Figure S1. ^{1}H NMR Spectrum (500 MHz) of tanzawaic acid R (**1**) in Methanol-d_4………...S4
Figure S2. ^{13}C NMR Spectrum (125 MHz) of tanzawaic acid R (**1**) in Methanol-d_4………..S4
Figure S3. HMQC Spectrum of tanzawaic acid R (**1**)……………………………….…S5
Figure S4. COSY Spectrum of tanzawaic acid R(**1**)…………………………...……….S5
Figure S5. HMBC Spectrum of tanzawaic acid R (**1**) ……………………………………S6
Figure S6. NOESY Spectrum of tanzawaic acid R (**1**) ………………………………..….S6
Figure S7. HRESIMS Spectrum of tanzawaic acid R (**1**) ………………………….…..S7
Figure S8. ^{1}H NMR Spectrum (500 MHz) of tanzawaic acid S (**2**) in Methanol-d_4…..……S7
Figure S9. ^{13}C NMR Spectrum (125 MHz) of tanzawaic acid S (**2**) in Methanol-d_4………..S8
Figure S10. HMQC Spectrum of tanzawaic acid S (**2**)…………………………………....S8
Figure S11. COSY Spectrum of tanzawaic acid S (**2**)……………………………………S9
Figure S12. HMBC Spectrum of tanzawaic acid S (**2**)…………………………………...S9
Figure S13. NOESY Spectrum of tanzawaic acid S (**2**)…………………………………S10
Figure S14. HRESIMS Spectrum of tanzawaic acid S (**2**)………………………………S10
Figure S15. ^{1}H NMR Spectrum (500 MHz) of tanzawaic acid T (**3**) in $CDCl_3$…...………..S11
Figure S16. ^{13}C NMR Spectrum (125 MHz) of tanzawaic acid T (**3**) in $CDCl_3$……………S11
Figure S17. HMQC Spectrum of tanzawaic acid T (**3**)…………………………………S12
Figure S18. COSY Spectrum of tanzawaic acid T (**3**)…………………………………..S12
Figure S19. HMBC Spectrum of tanzawaic acid T (**3**)…………………………………S13
Figure S20. NOESY Spectrum of tanzawaic acid T (**3**) ………………………………..S13
Figure S21. HRESIMS Spectrum of tanzawaic acid T (**3**) ……………………………..S14
Figure S22. ^{1}H NMR Spectrum (500 MHz) of tanzawaic acid U (**4**) in $CDCl_3$…………..S14
Figure S23. ^{13}C NMR Spectrum (125 MHz) of tanzawaic acid U (**4**) in $CDCl_3$…………S15
Figure S24. HMQC Spectrum of tanzawaic acid U (**4**) ………………………………..S15
Figure S25. COSY Spectrum of tanzawaic acid U (**4**) …………………………………S16
Figure S26. HMBC Spectrum of tanzawaic acid U (**4**) ………………………………..S16
Figure S27. NOESY Spectrum of tanzawaic acid U (**4**) ………………………………S17
Figure S28. HRESIMS Spectrum of tanzawaic acid U (**4**) ……………………………S17
Figure S29. ^{1}H NMR Spectrum (500 MHz) of tanzawaic acid V (**5**) in Methanol-d_4…...S18
Figure S30. ^{13}C NMR Spectrum (125 MHz) of tanzawaic acid V (**5**) in Methanol-d_4…...S18
Figure S31. HMQC Spectrum of tanzawaic acid V (**5**) ………………………………S19
Figure S32. COSY Spectrum of tanzawaic acid V (**5**) ……………………….……….S19
Figure S33. HMBC Spectrum of tanzawaic acid V (**5**) ………………………………S20
Figure S34. NOESY Spectrum of tanzawaic acid V (**5**) ………………………………S20
Figure S35. HRESIMS Spectrum of tanzawaic acid V (**5**) …………………………..S21
Figure S36. ^{1}H NMR Spectrum (500 MHz) of tanzawaic acid W (**6**) in $CDCl_3$………..S21
Figure S37. ^{13}C NMR Spectrum (125 MHz) of tanzawaic acid W (**6**) in $CDCl_3$……….S22
Figure S38. HMQC Spectrum of tanzawaic acid W (**6**)………………………………S22
Figure S39. COSY Spectrum of tanzawaic acid W (**6**) ……………………...………S23
Figure S40. HMBC Spectrum of tanzawaic acid W (**6**)………………………………S23
Figure S41. NOESY Spectrum of tanzawaic acid W (**6**)……………….……….….…S24
Figure S42. HRESIMS Spectrum of tanzawaic acid W (**6**) ……………………..….S24
Figure S43. ^{1}H NMR Spectrum (500 MHz) of tanzawaic acid X (**11**) in $CDCl_3$………...S25

Figure S44. ^{13}C NMR Spectrum (125 MHz) of tanzawaic acid X (**11**) in $CDCl_3$…………S25
Figure S45. HMQC Spectrum of tanzawaic acid X (**11**)……………………….……..S26
Figure S46. COSY Spectrum of tanzawaic acid X (**11**)……………………….………S26
Figure S47. HMBC Spectrum of tanzawaic acid X (**11**)…………………….………..S27
Figure S48. NOESY Spectrum of tanzawaic acid X (**11**)………………….……………S27
Figure S49. HRESIMS Spectrum of tanzawaic acid X (**11**) ………………………………S28
Figure S50. ^{1}H NMR Spectrum (500 MHz) of **1a** in Methanol-d_4…………………………S28
Figure S51. ^{1}H NMR Spectrum (500 MHz) of **1b** in Methanol-d_4…………………………S29
Figure S52. ^{13}C NMR Spectrum (125 MHz) of **1b** in Methanol-d_4………………………..S29
Figure S53. ^{1}H NMR Spectrum (500 MHz) of **12a** in $CDCl_3$…………………………...S30
Figure S54. ^{13}C NMR Spectrum (125 MHz) of **12a** in $CDCl_3$……………………………S30
Figure S55. IR spectrum of tanzawaic acid R (**1**)…………………………………………S31
Figure S56. IR spectrum of tanzawaic acid S (**2**)…………………………….………S31
Figure S57. IR spectrum of tanzawaic acid T (**3**)…………………………………………S31
Figure S58. IR spectrum of tanzawaic acid U (**4**)…………………………………………S32
Figure S59. IR spectrum of tanzawaic acid V (**5**)…………………………………………S32
Figure S60. IR spectrum of tanzawaic acid W (**6**)…………………………………………S32
Figure S61. IR spectrum of tanzawaic acid X (**11**)……………………………………...…S33

Figure S1. ^{1}H NMR Spectrum (500 MHz) of tanzawaic acid R (**1**) in Methanol-d_4

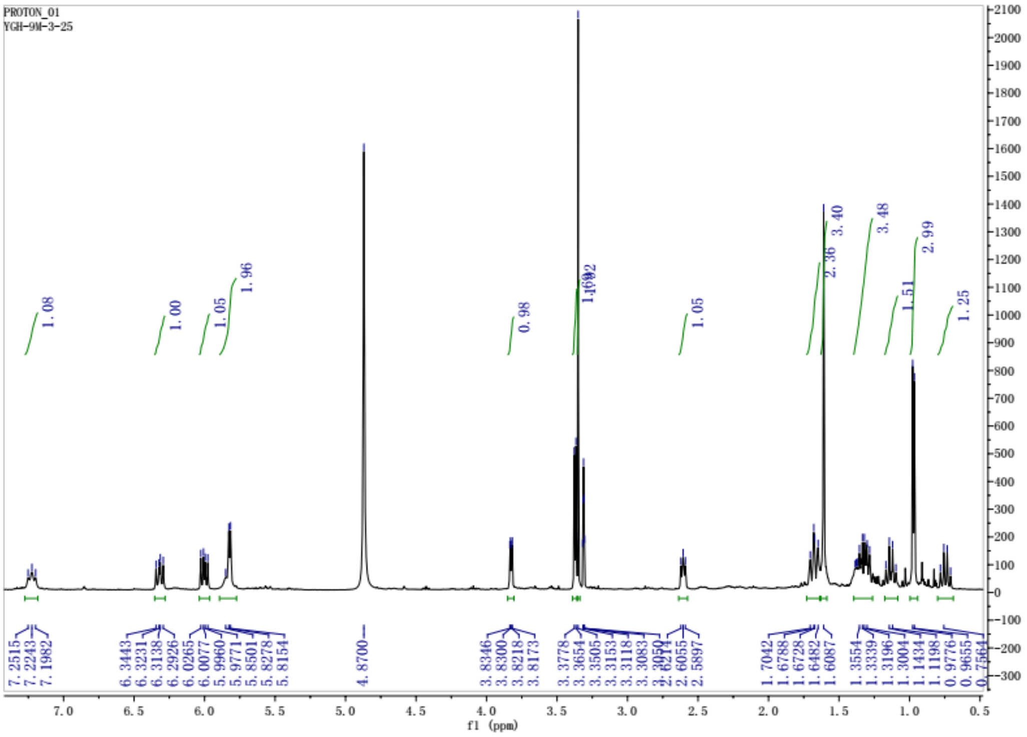

Figure S2. ^{13}C NMR Spectrum (125 MHz) of tanzawaic acid R (**1**) in Methanol-d_4

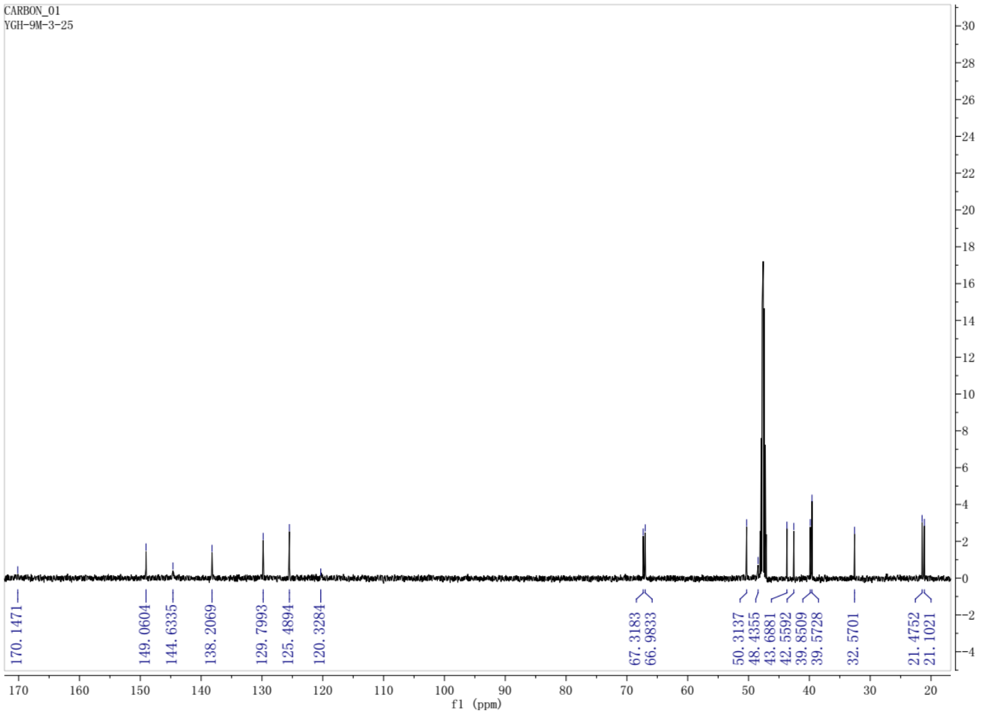

Figure S3. HMQC Spectrum of tanzawaic acid R (**1**)

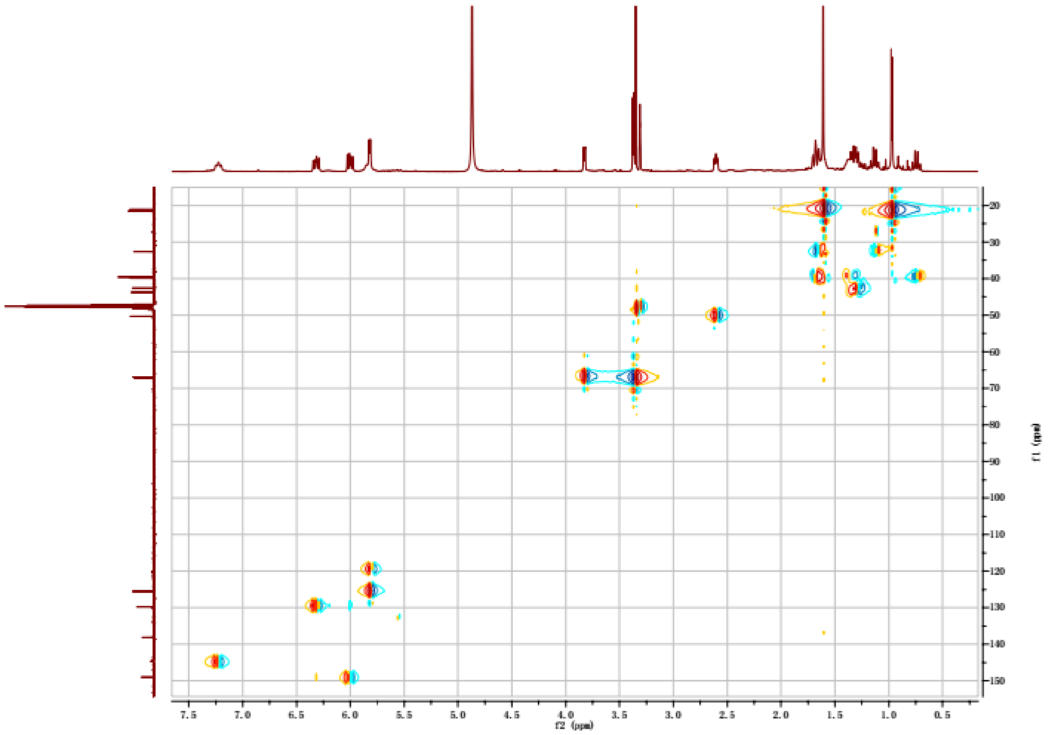

Figure S4. COSY Spectrum of tanzawaic acid R (**1**)

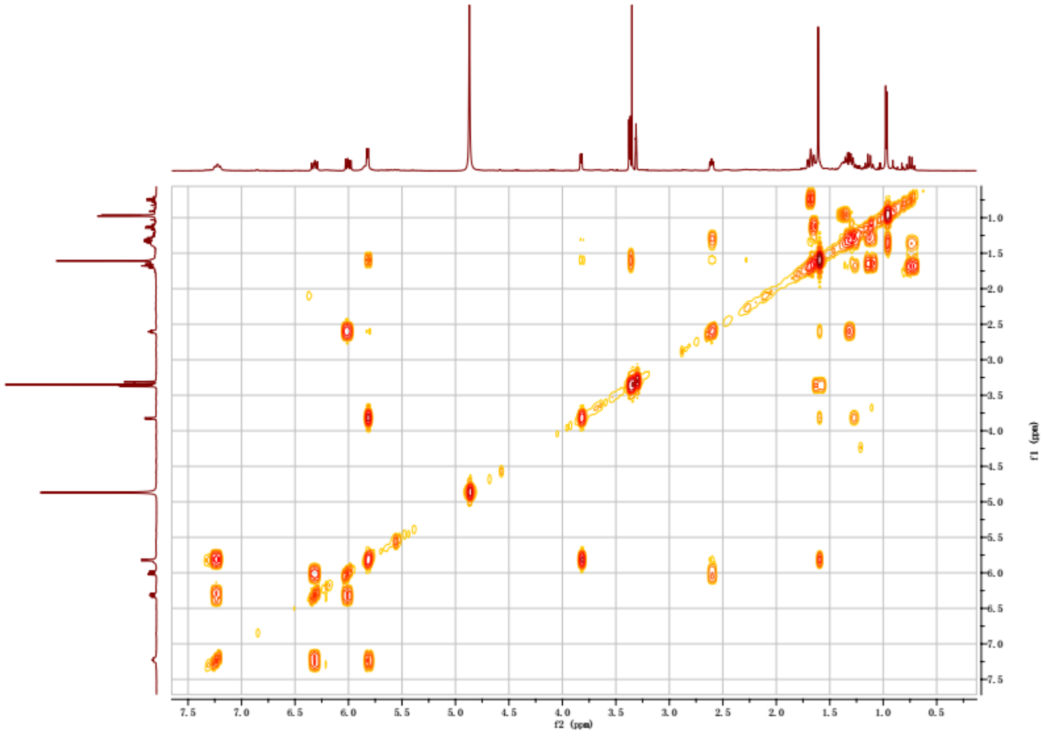

Figure S5. HMBC Spectrum of tanzawaic acid R (**1**)

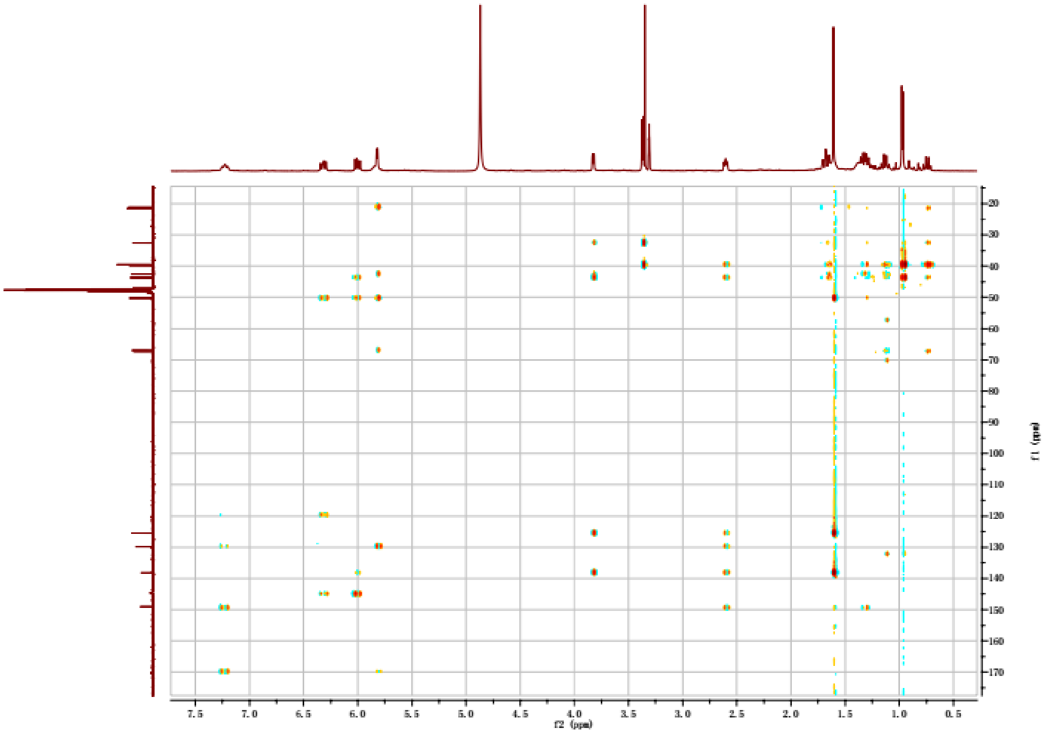

Figure S6. NOESY Spectrum of tanzawaic acid R (**1**)

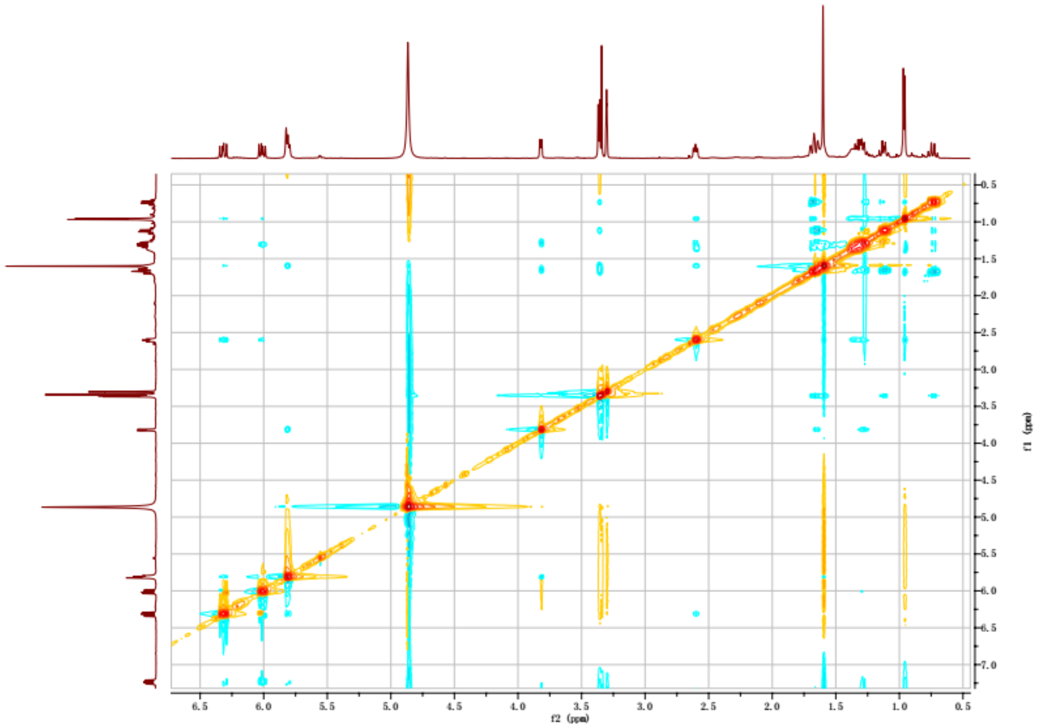

Figure S7. HRESIMS Spectrum of tanzawaic acid R (**1**)

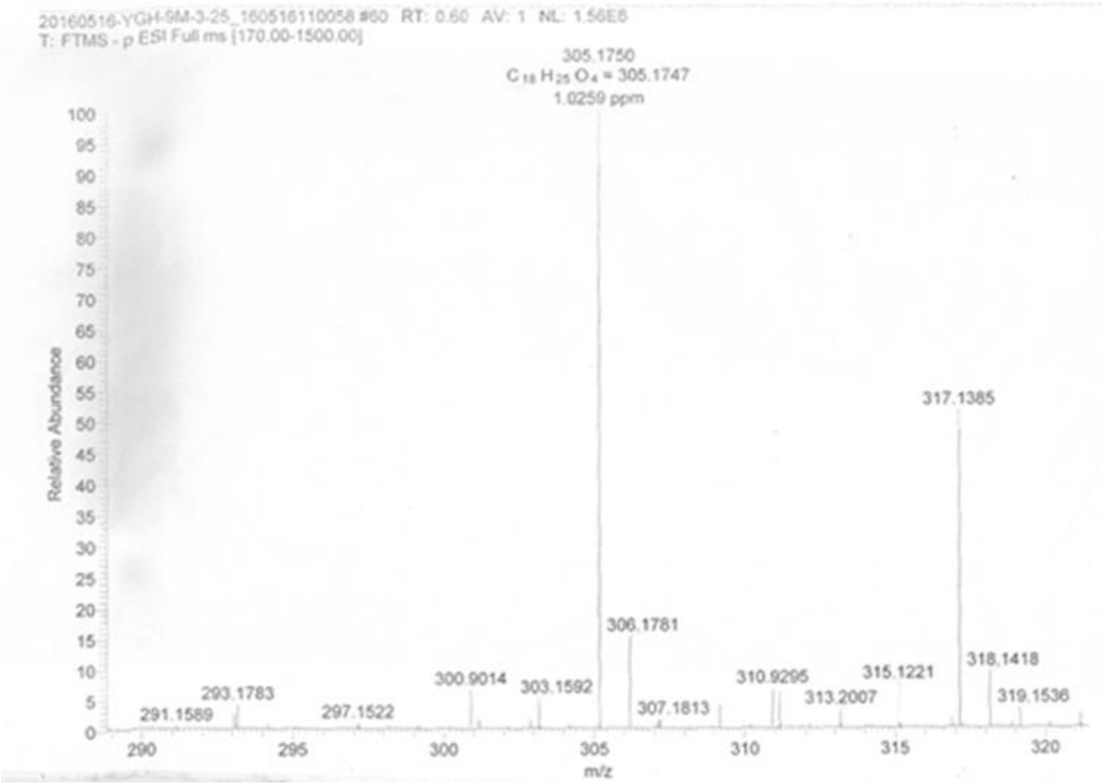

Figure S8. ^{1}H NMR Spectrum (500 MHz) of tanzawaic acid S (**2**) in Methanol-d_4

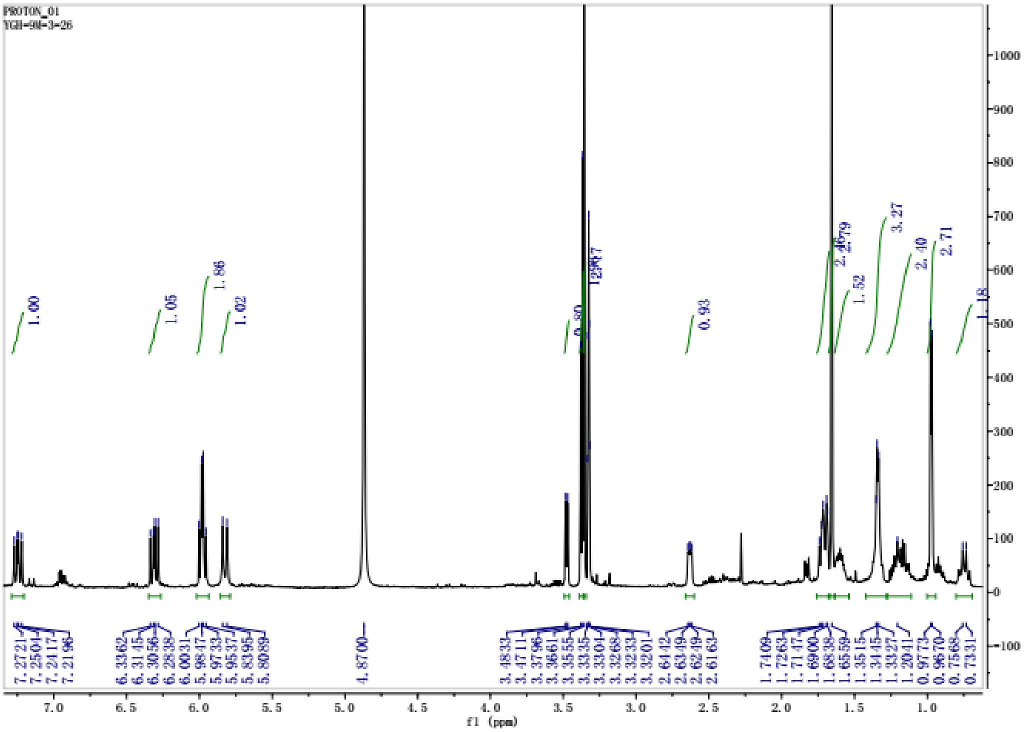

Figure S9. ^{13}C NMR Spectrum (125 MHz) of tanzawaic acid S (**2**) in Methanol-d_4

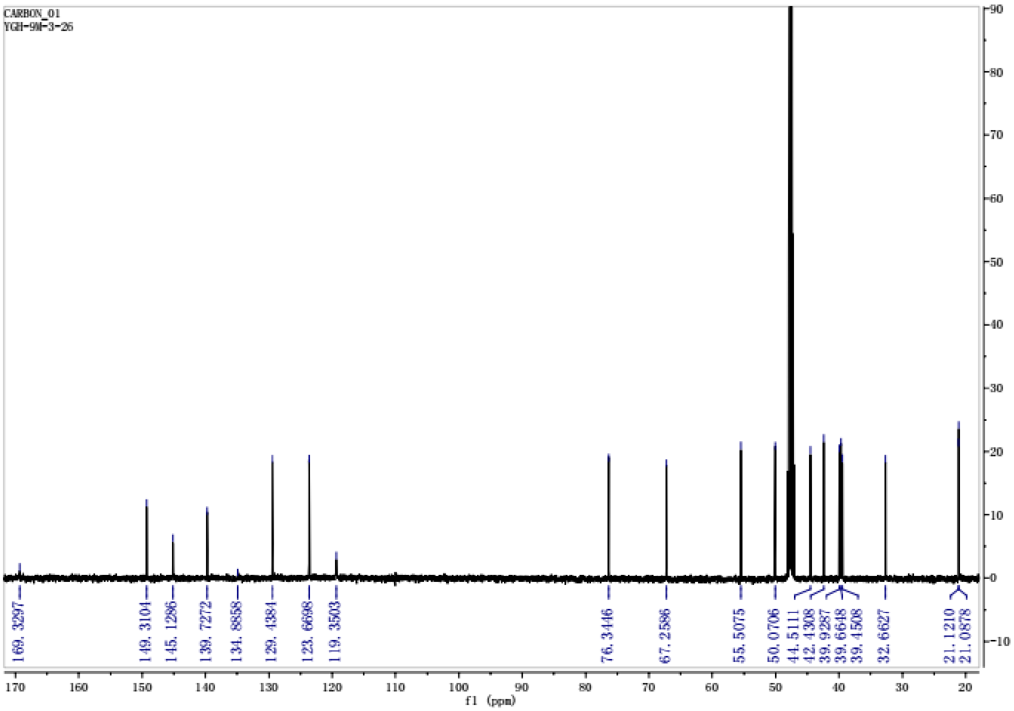

Figure S10. HMQC Spectrum of tanzawaic acid S (**2**)

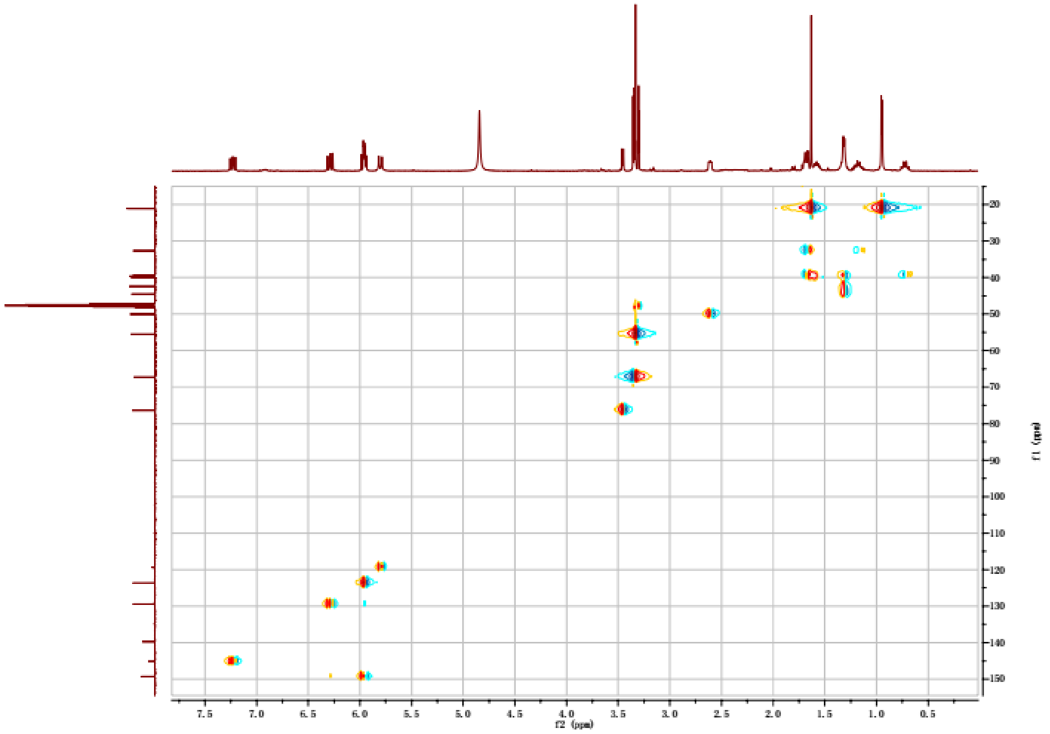

Figure S11. COSY Spectrum of tanzawaic acid S (**2**)

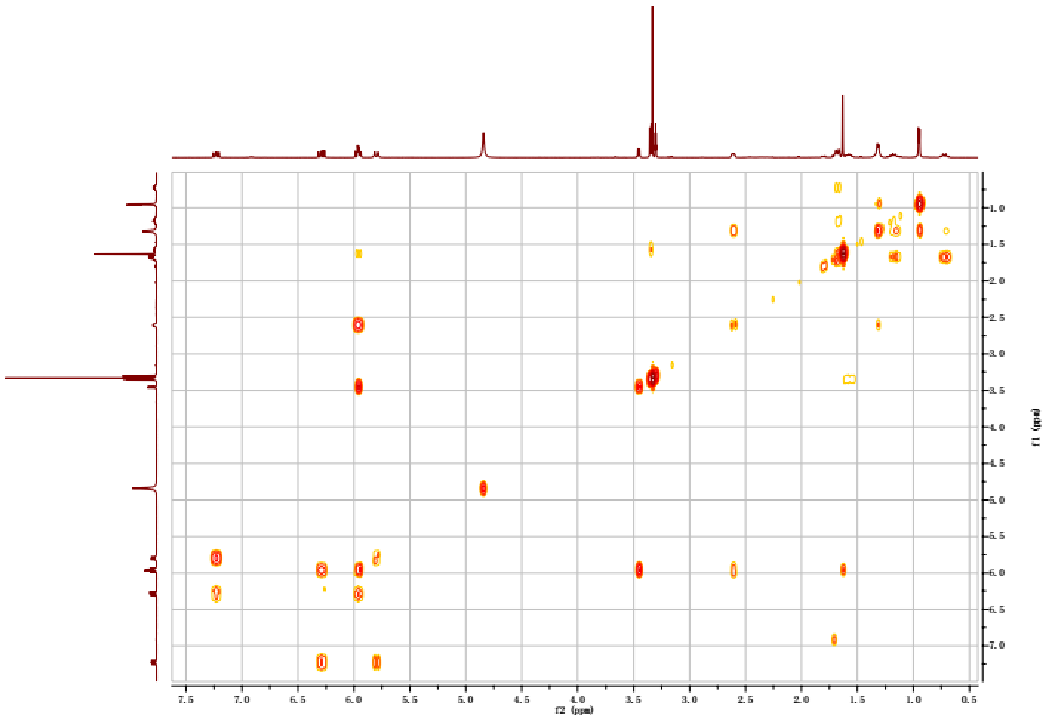

Figure S12. HMBC Spectrum of tanzawaic acid S (**2**)

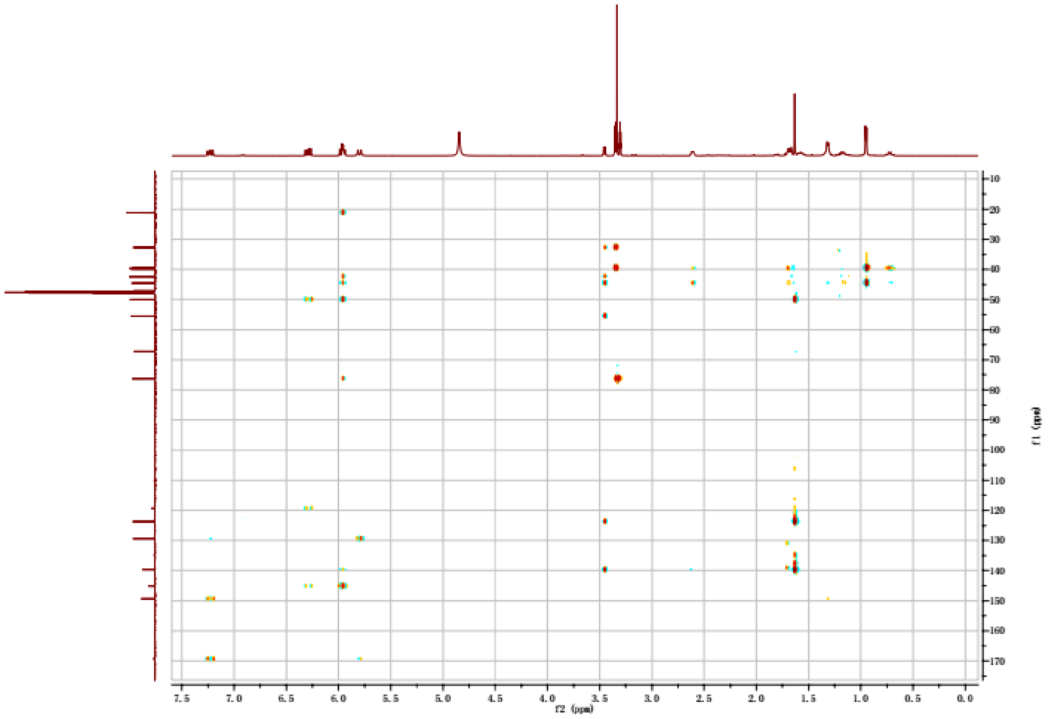

Figure S13. NOESY Spectrum of tanzawaic acid S (**2**)

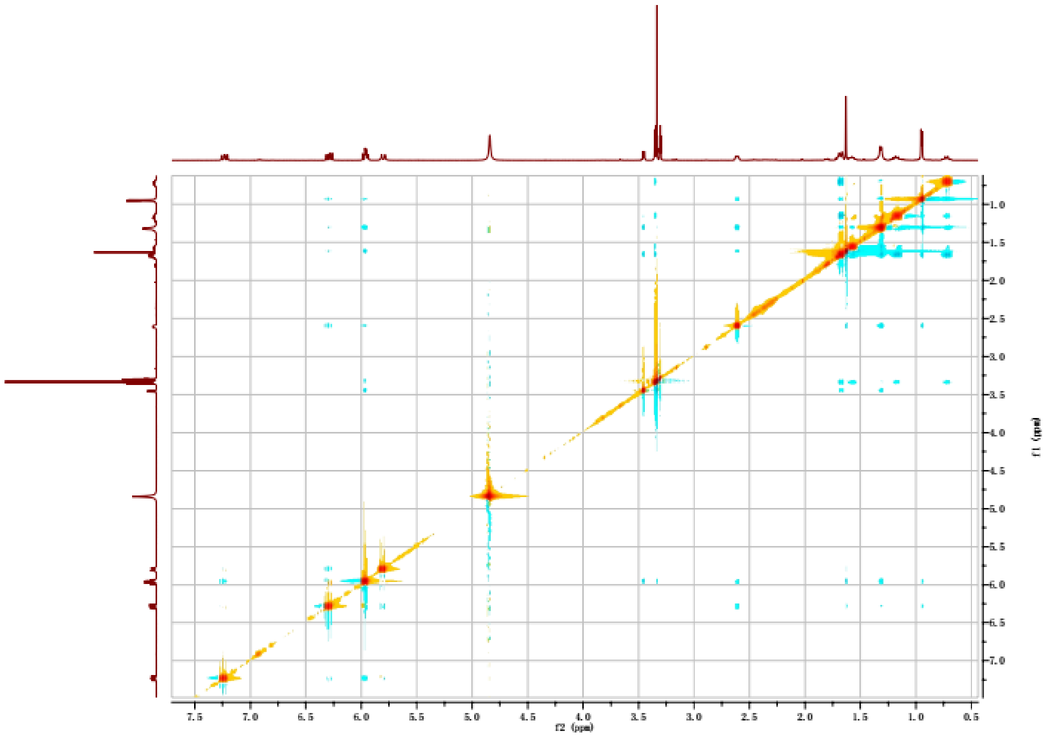

Figure S14. HRESIMS Spectrum of tanzawaic acid S (**2**)

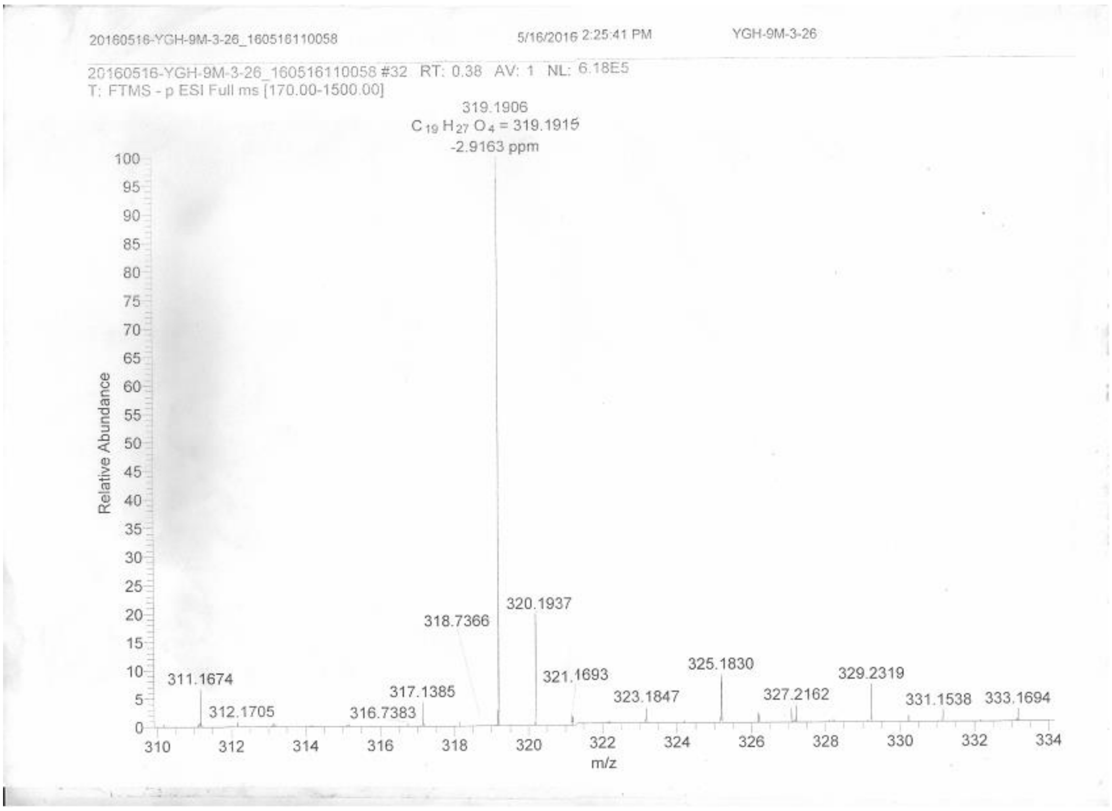

Figure S15. ^{1}H NMR Spectrum (500 MHz) of tanzawaic acid T (**3**) in $CDCl_3$

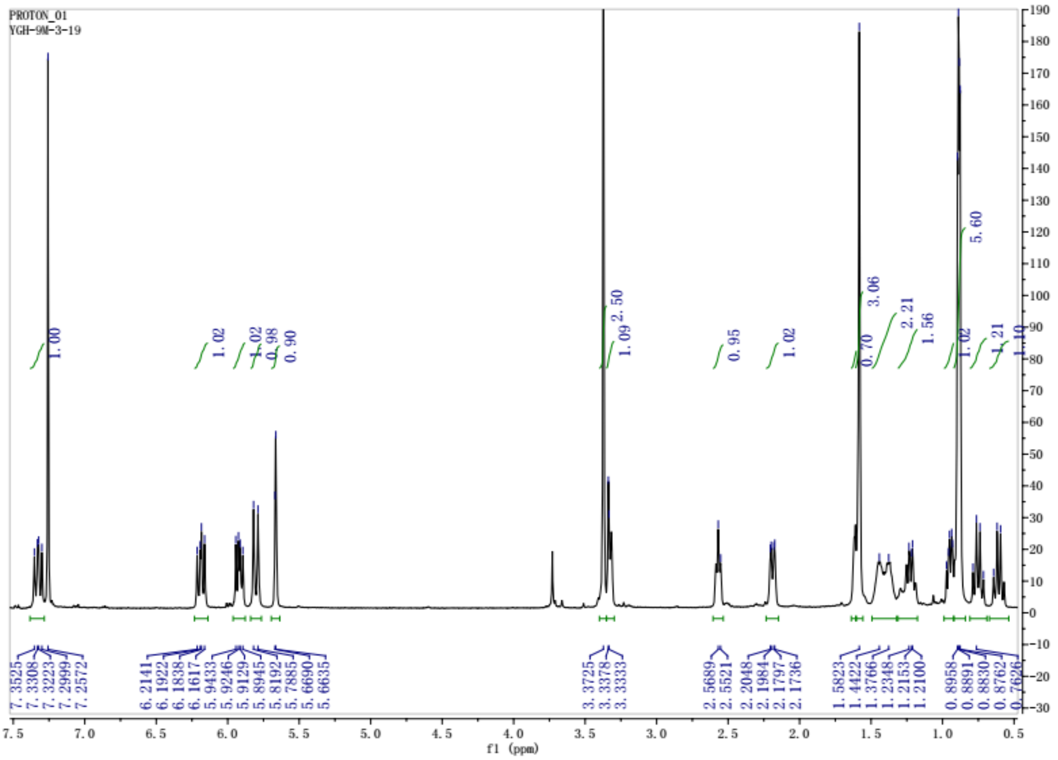

Figure S16. ^{13}C NMR Spectrum (125 MHz) of tanzawaic acid T (**3**) in $CDCl_3$

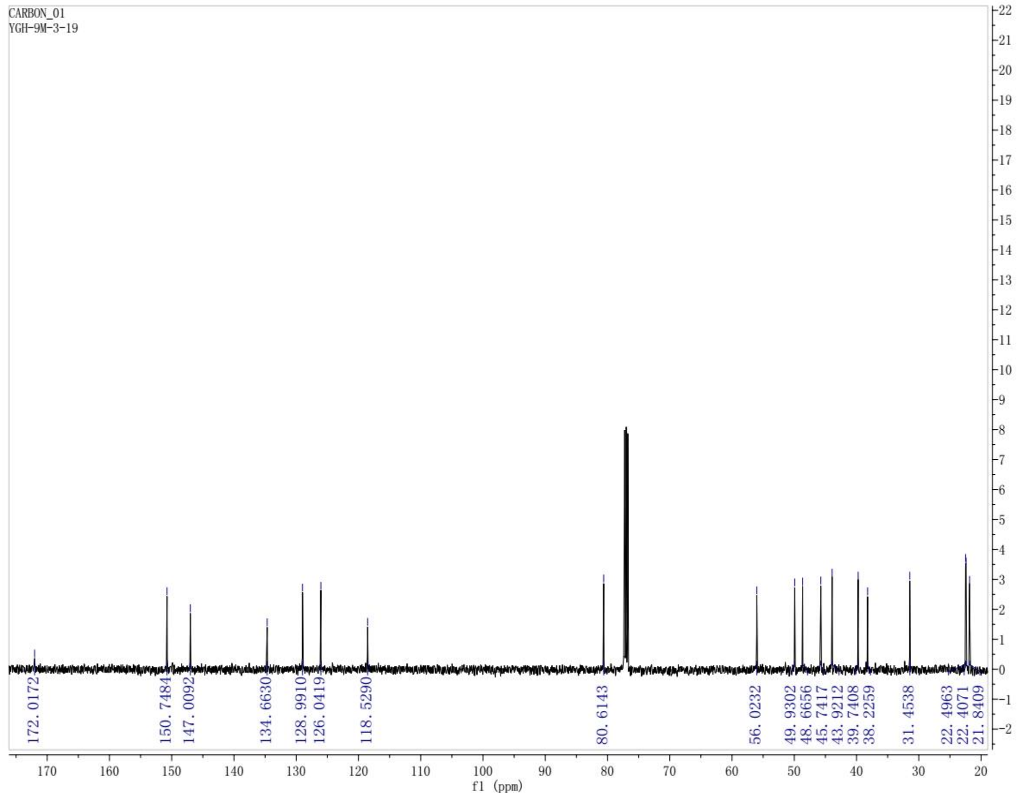

Figure S17. HMQC Spectrum of tanzawaic acid T (**3**)

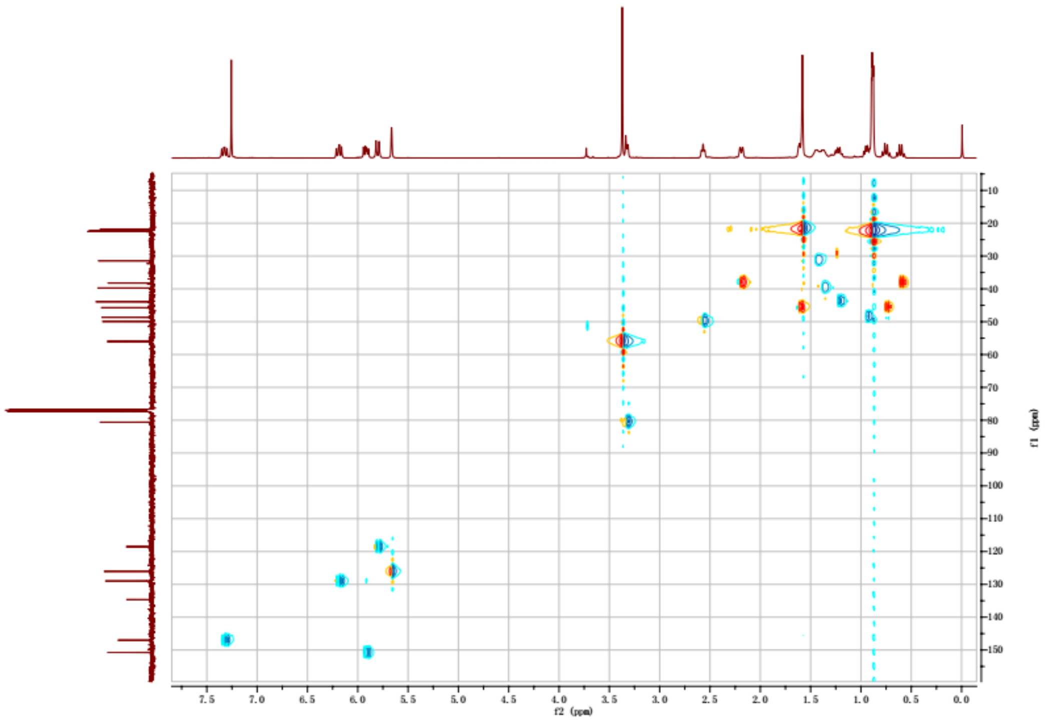

Figure S18. COSY Spectrum of tanzawaic acid T (**3**)

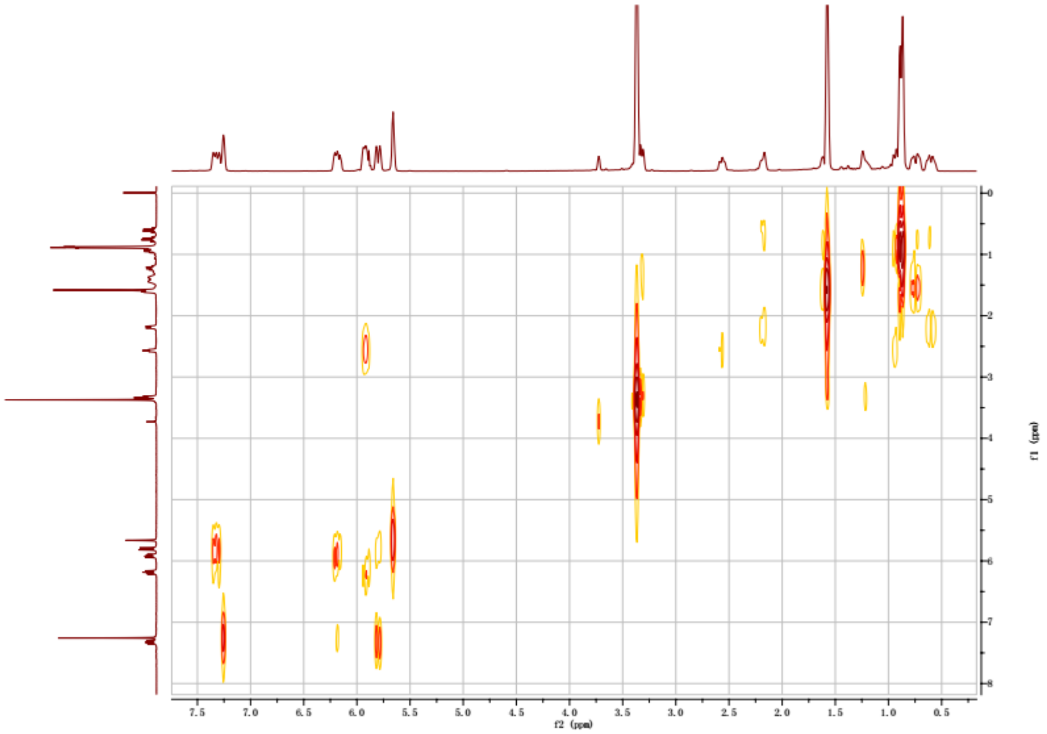

Figure S19. HMBC Spectrum of tanzawaic acid T (**3**)

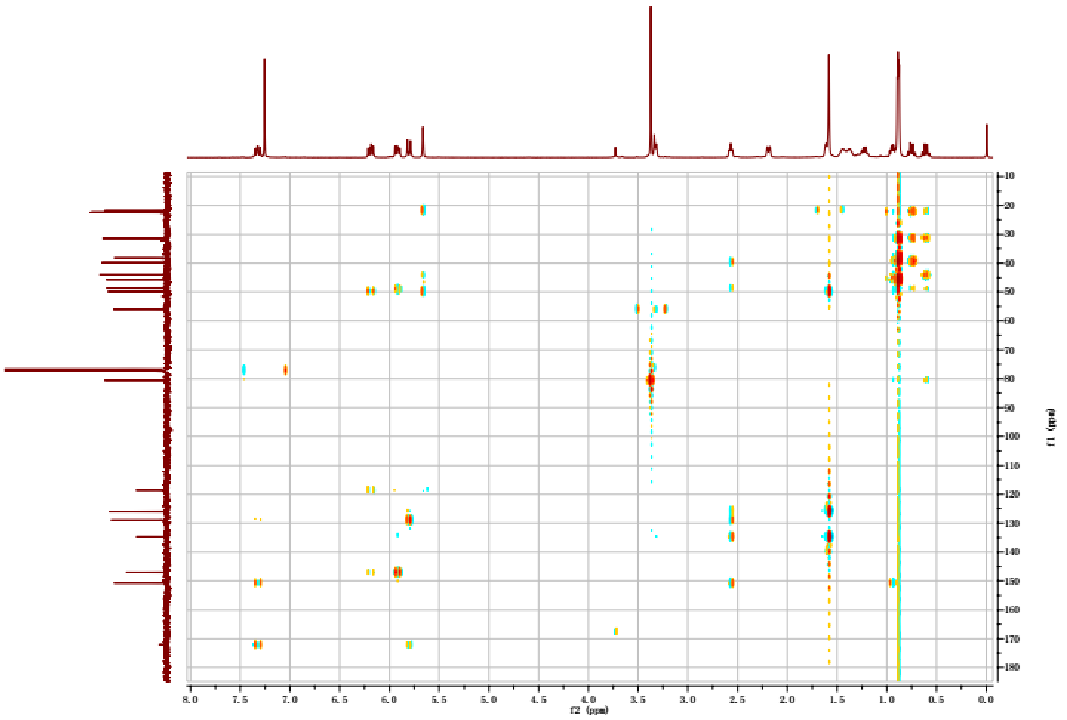

Figure S20. NOESY Spectrum of tanzawaic acid T (**3**)

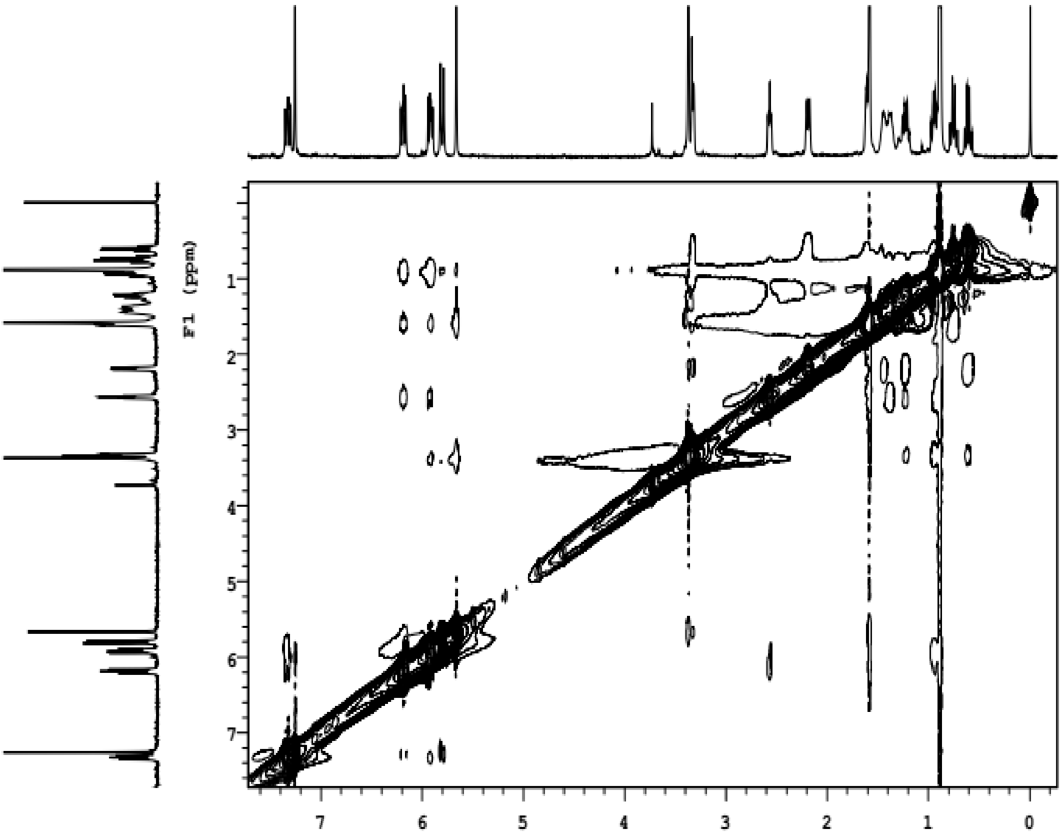

Figure S21. HRESIMS Spectrum of tanzawaic acid T (**3**)

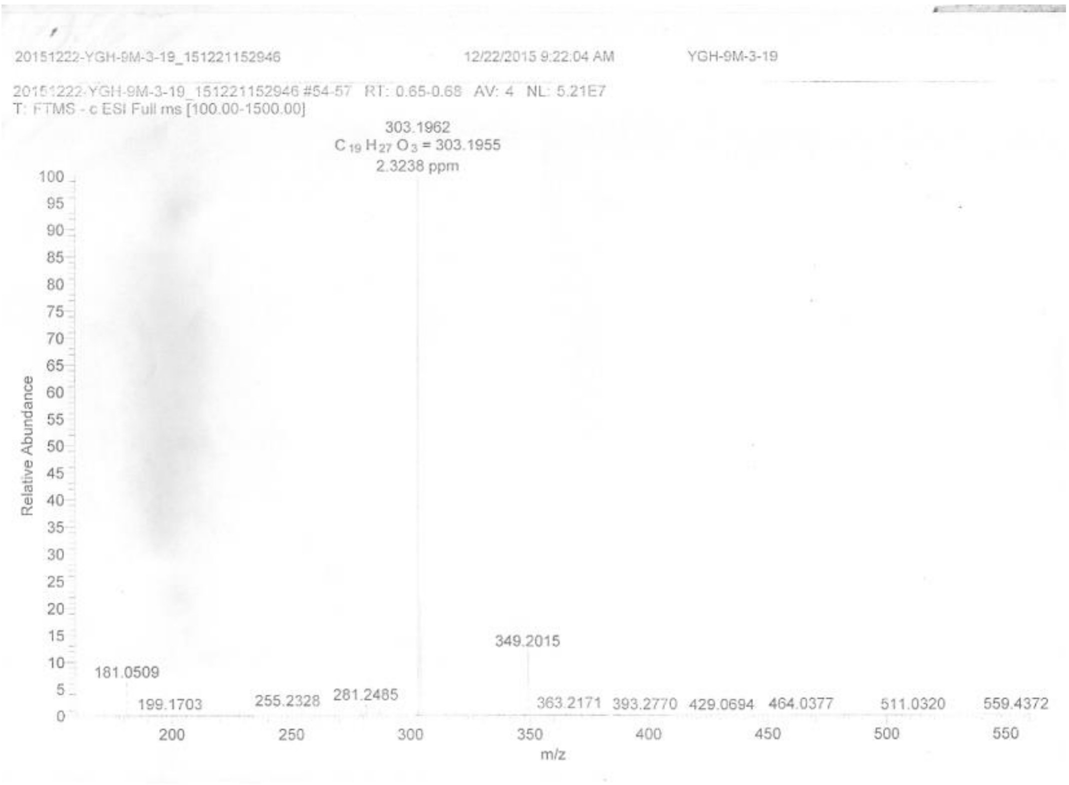

Figure S22. ^{1}H NMR Spectrum (500 MHz) of tanzawaic acid U (**4**) in $CDCl_3$

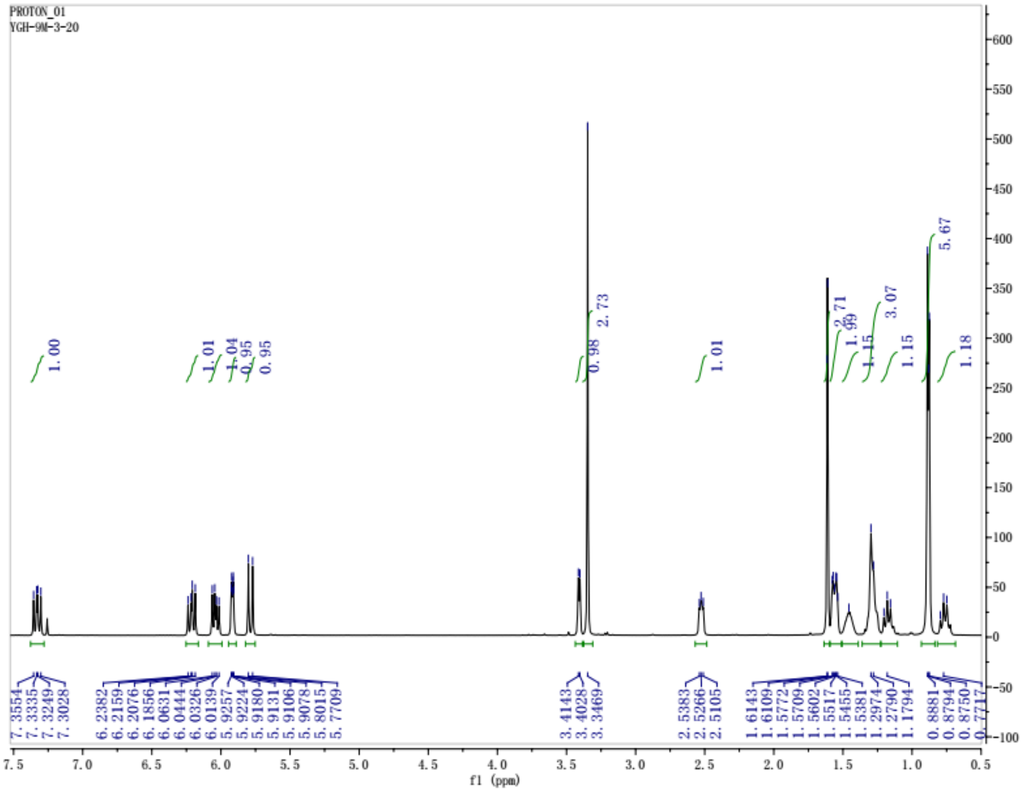

Figure S23. ^{13}C NMR Spectrum (125 MHz) of tanzawaic acid U (**4**) in $CDCl_3$

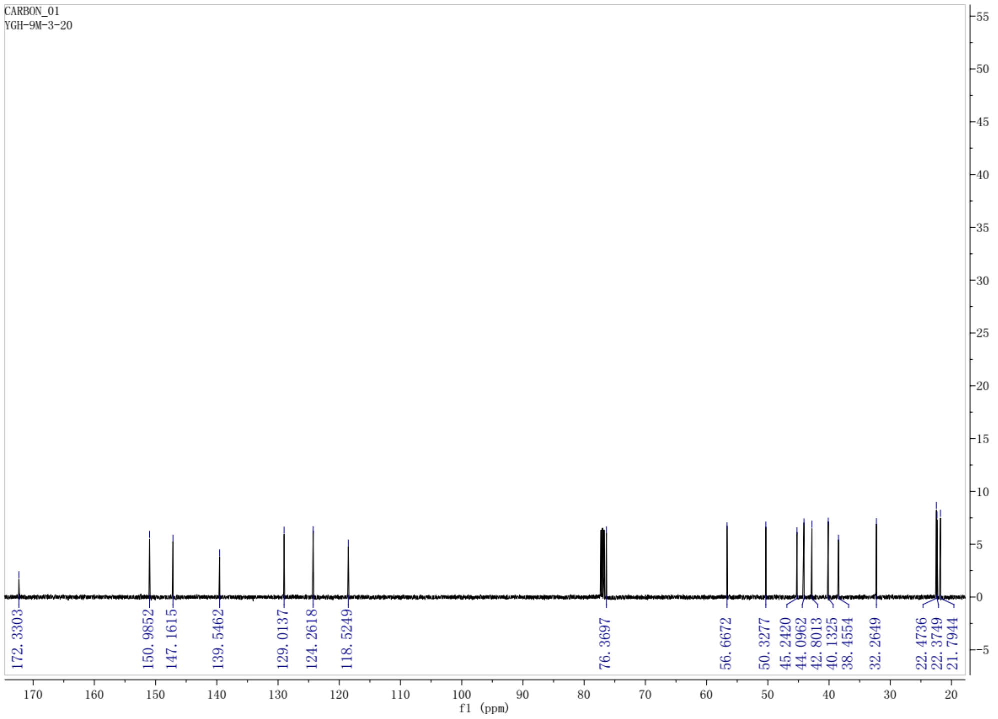

Figure S24. HMQC Spectrum of tanzawaic acid U (**4**)

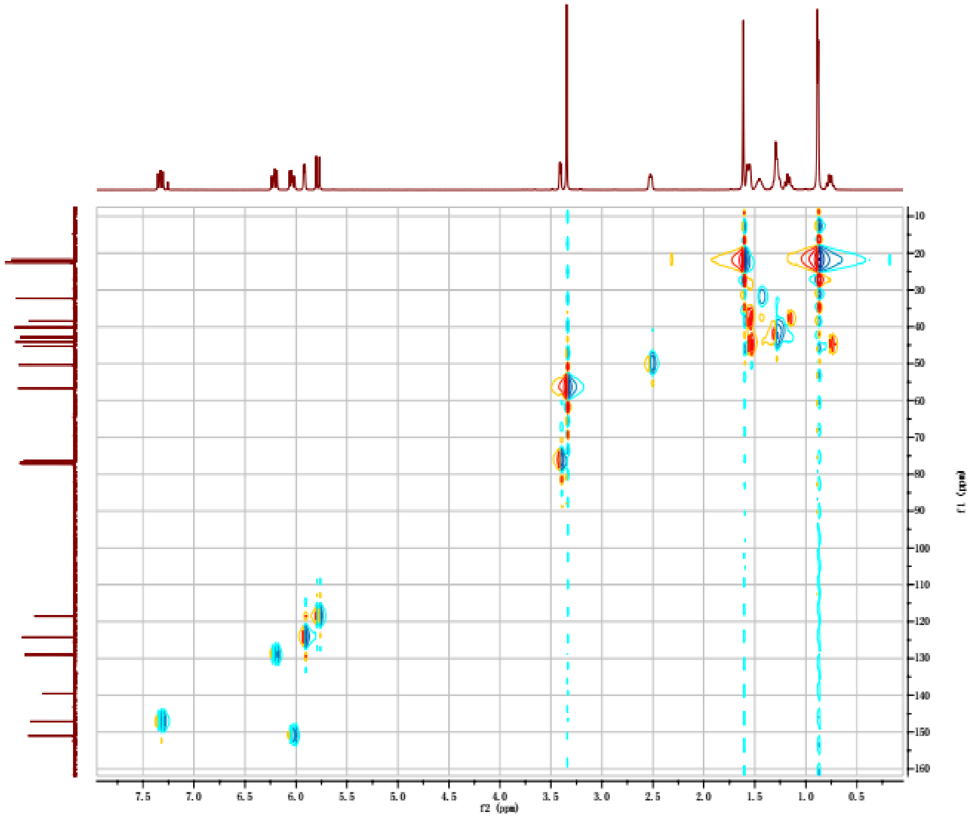

Figure S25. COSY Spectrum of tanzawaic acid U (**4**)

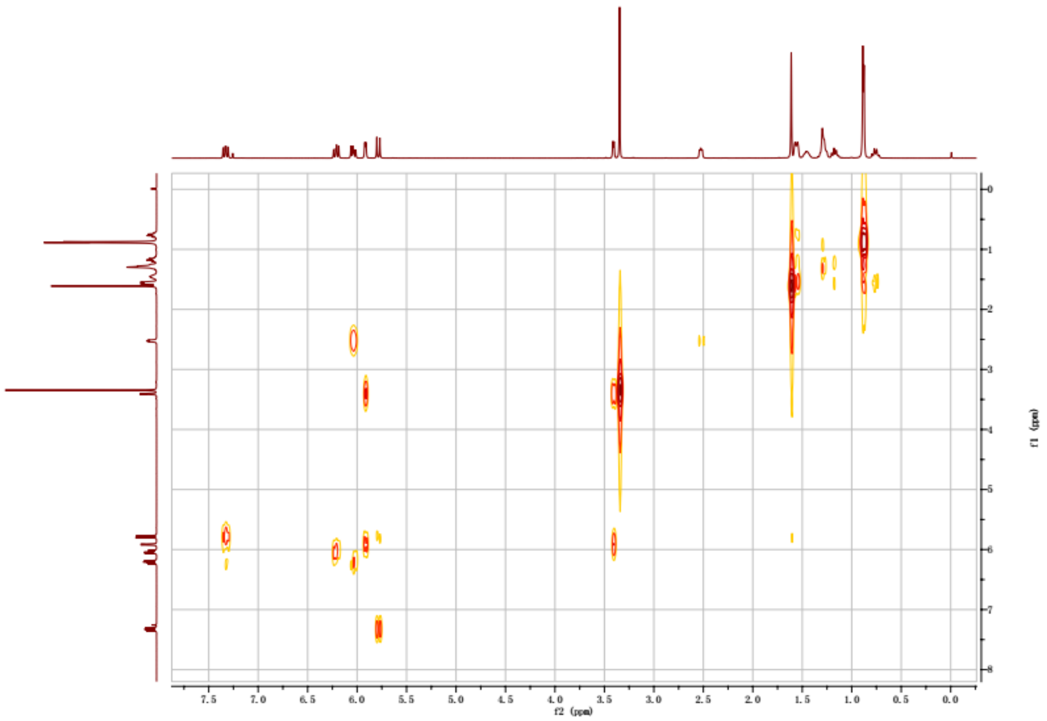

Figure S26. HMBC Spectrum of tanzawaic acid U (**4**)

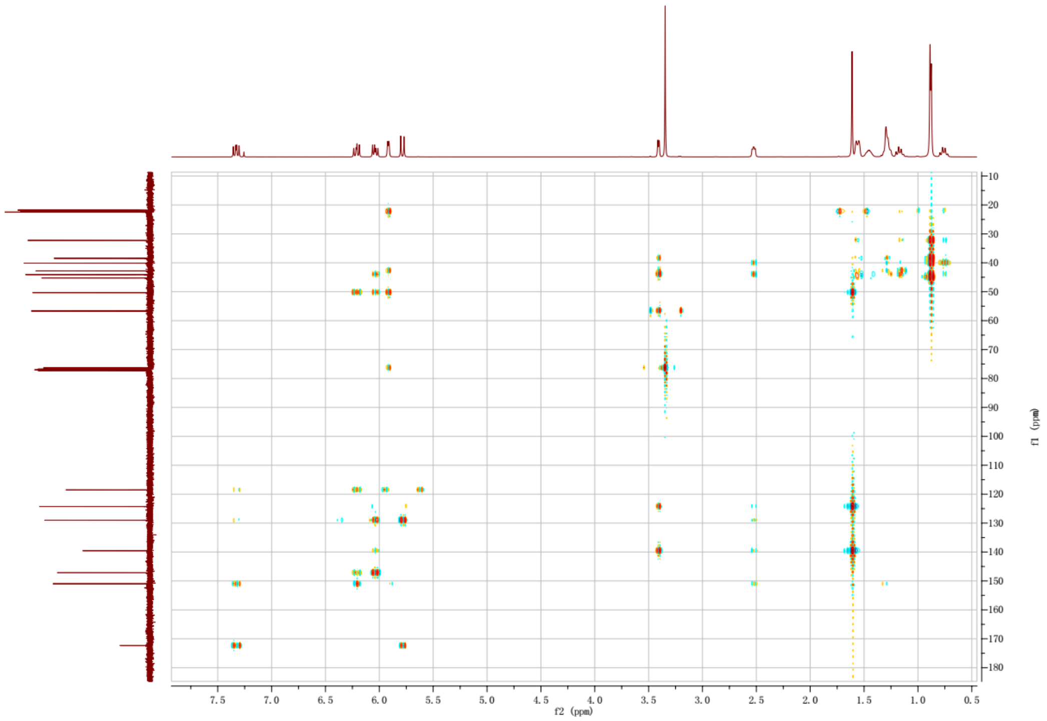

Figure S27. NOESY Spectrum of tanzawaic acid U (**4**)

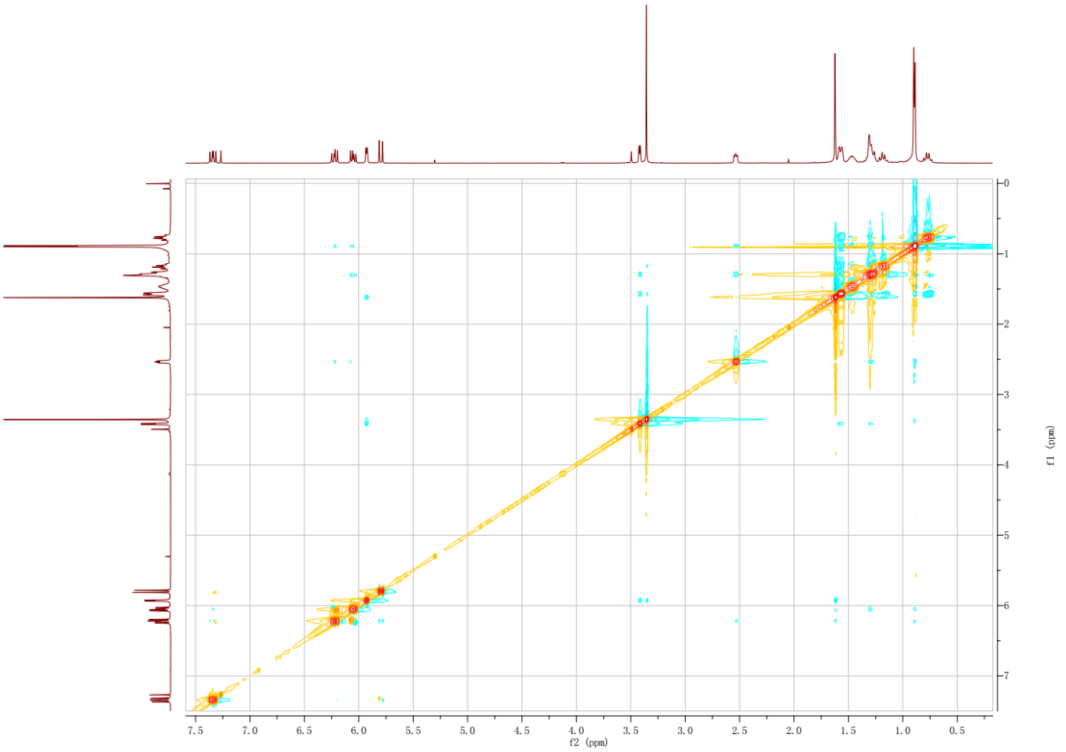

Figure S28. HRESIMS Spectrum of tanzawaic acid U (**4**)

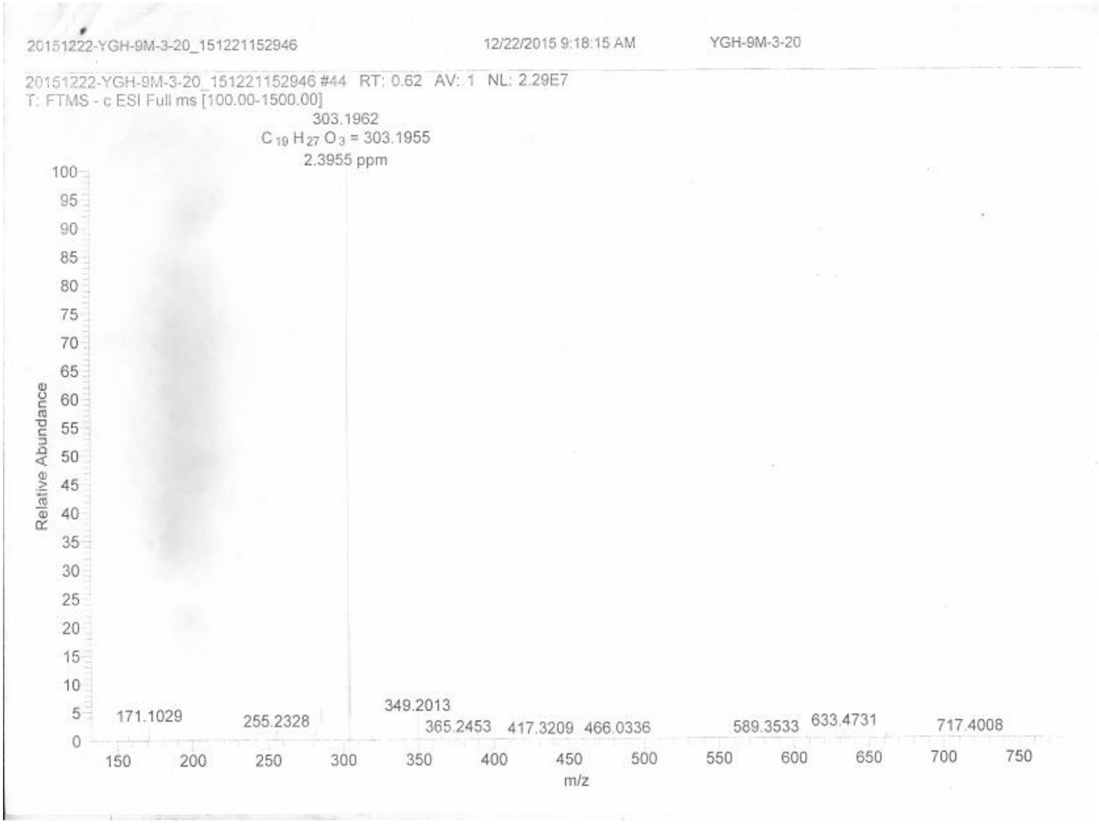

Figure S29. ^{1}H NMR Spectrum (500 MHz) of tanzawaic acid V (**5**) in Methanol-d_4

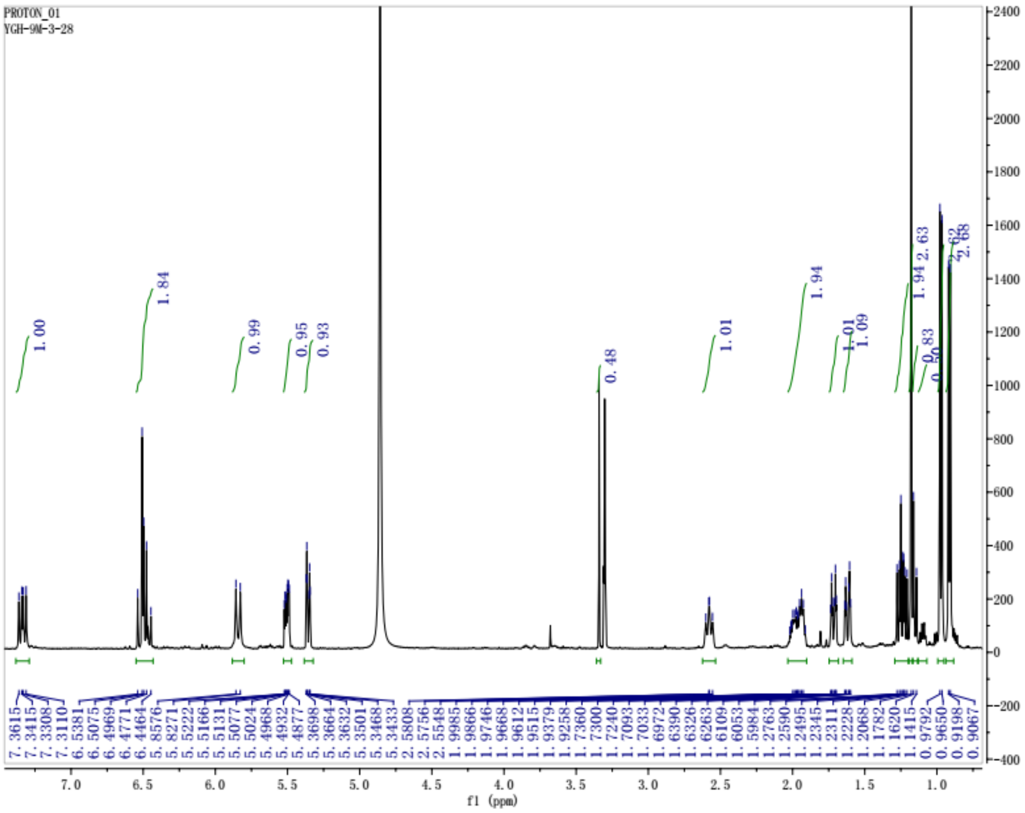

Figure S30. ^{13}C NMR Spectrum (125 MHz) of tanzawaic acid V (**5**) in Methanol-d_4

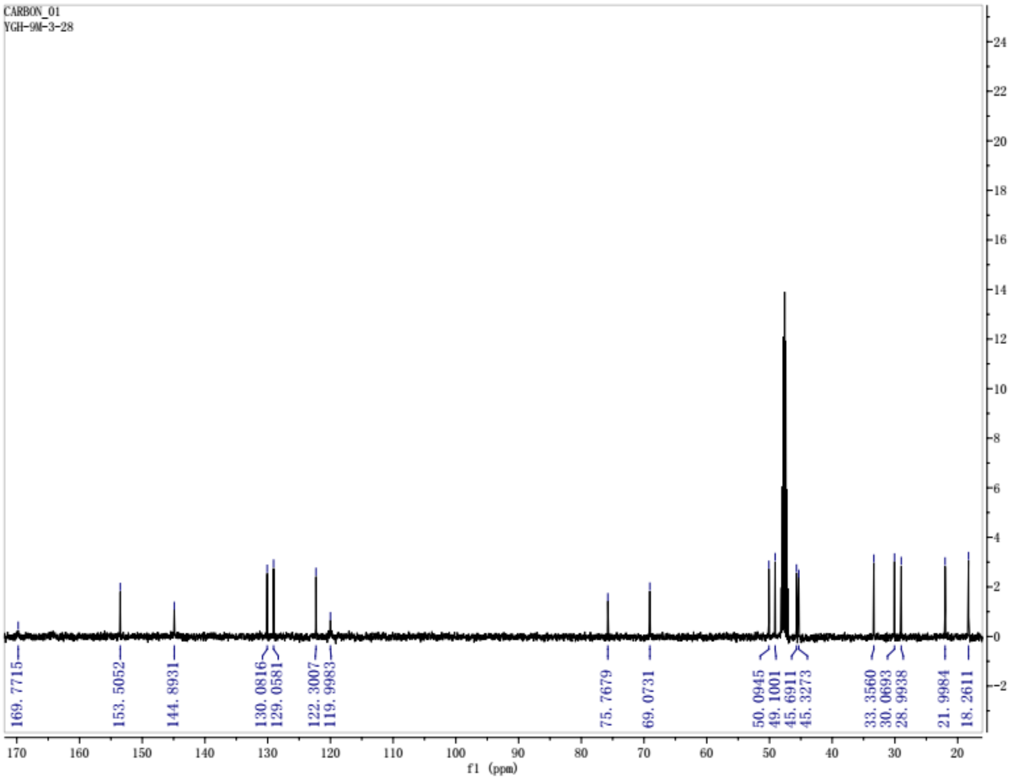

Figure S31. HMQC Spectrum of tanzawaic acid V (**5**)

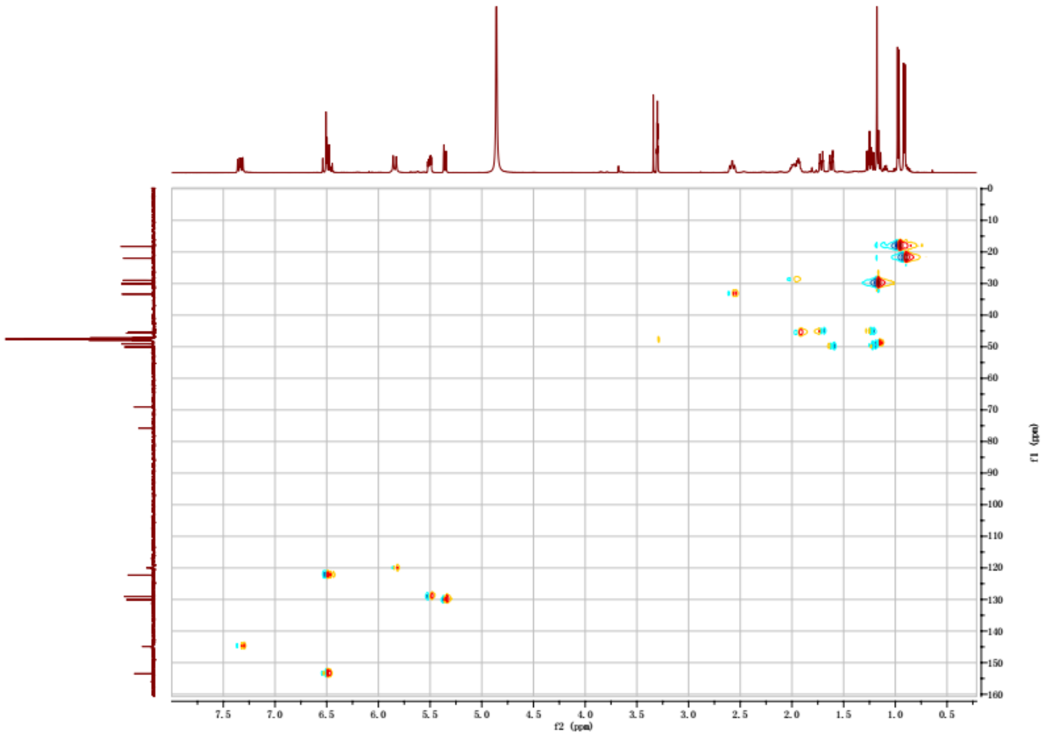

Figure S32. COSY Spectrum of tanzawaic acid V (**5**)

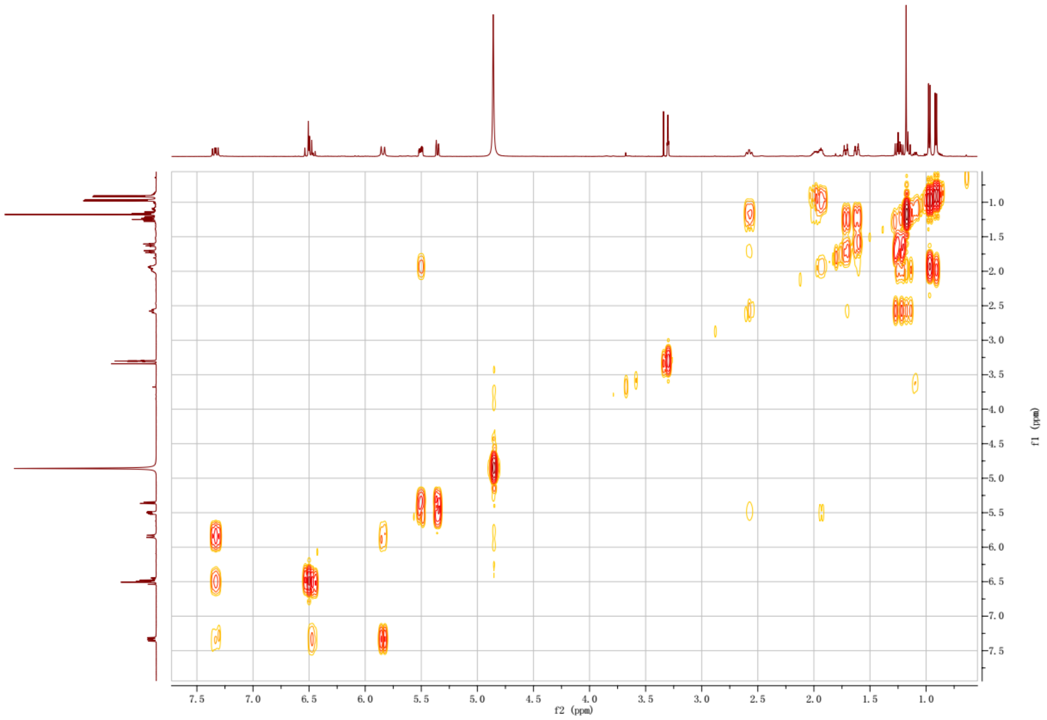

Figure S33. HMBC Spectrum of tanzawaic acid V (**5**)

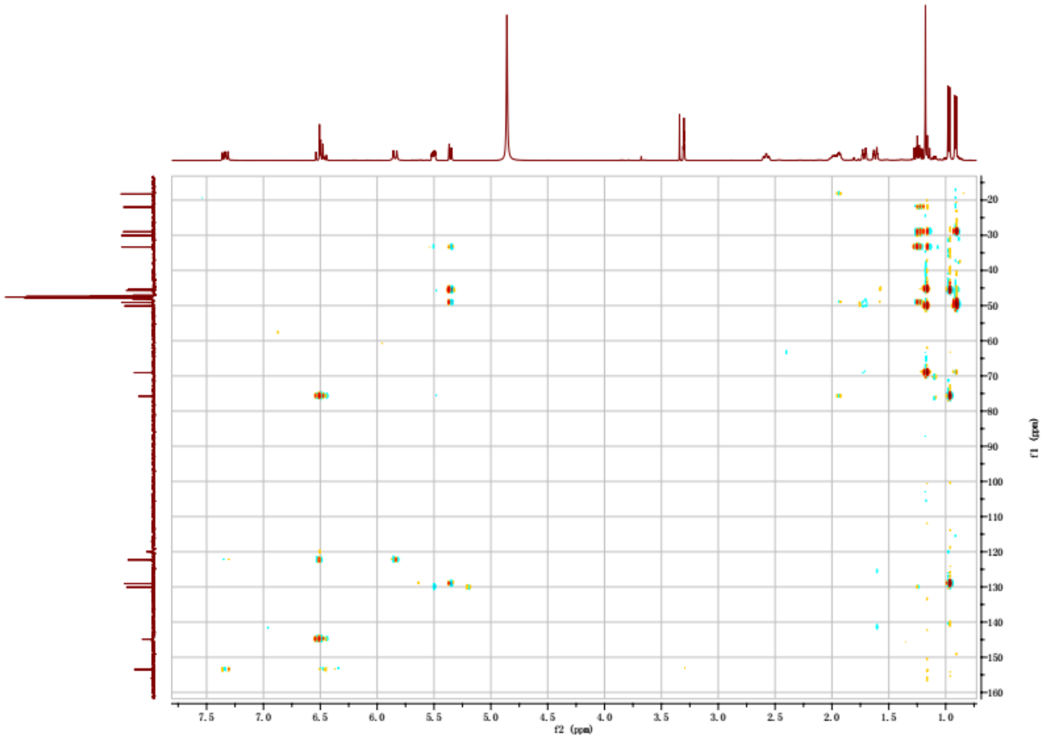

Figure S34. NOESY Spectrum of tanzawaic acid V (**5**)

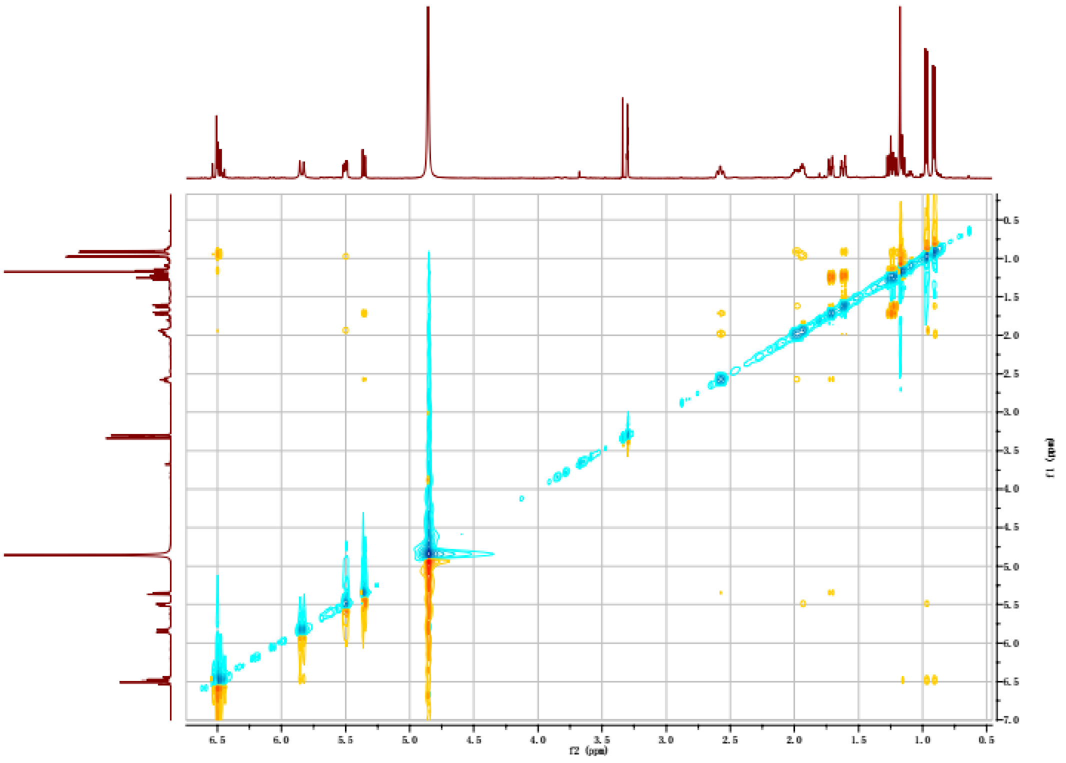

Figure S35. HRESIMS Spectrum of tanzawaic acid V (**5**)

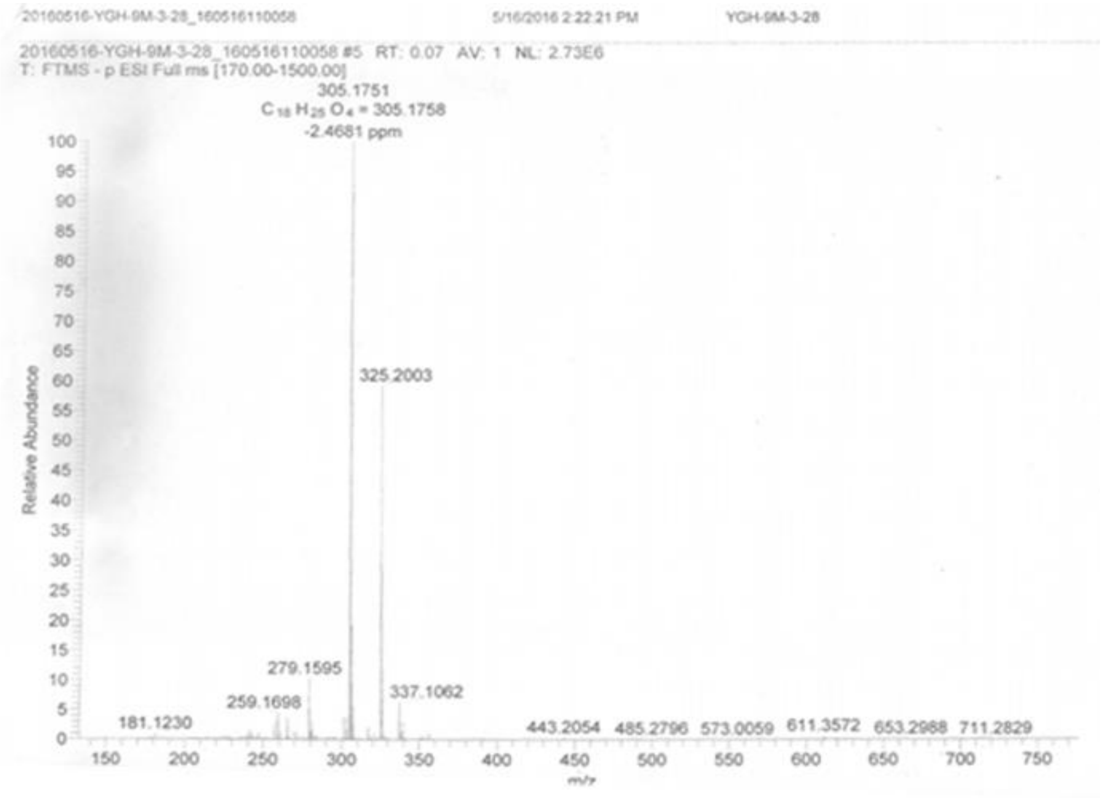

Figure S36. ^{1}H NMR Spectrum (500 MHz) of tanzawaic acid W (**6**) in $CDCl_3$

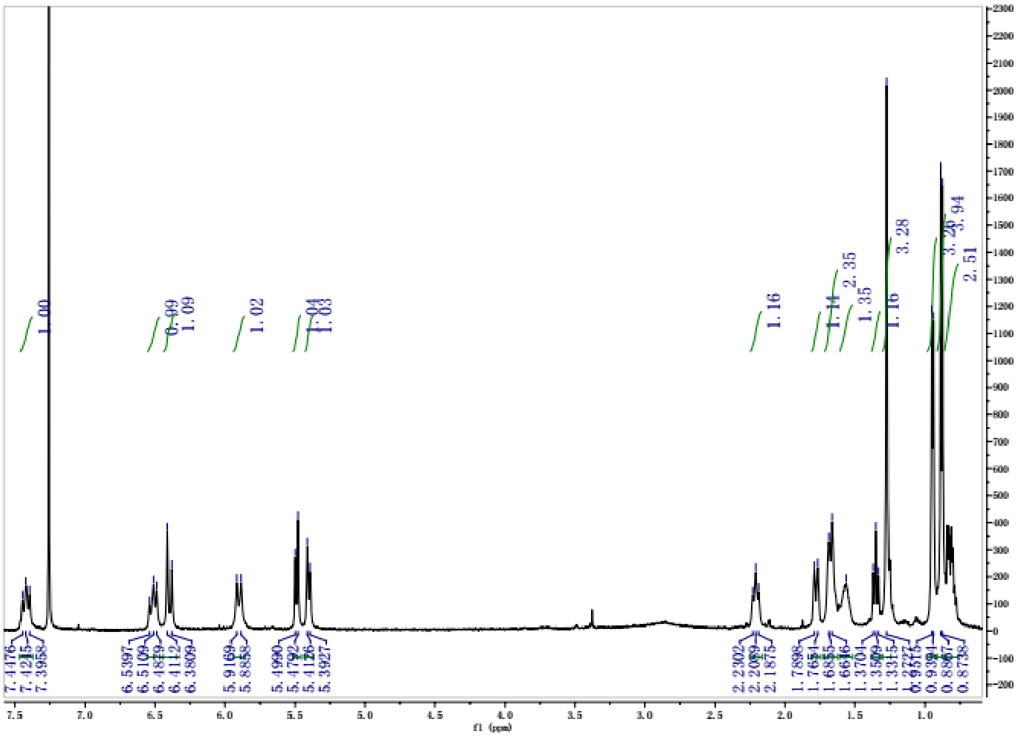

Figure S37. ^{13}C NMR Spectrum (125 MHz) of tanzawaic acid W (**6**) in $CDCl_3$

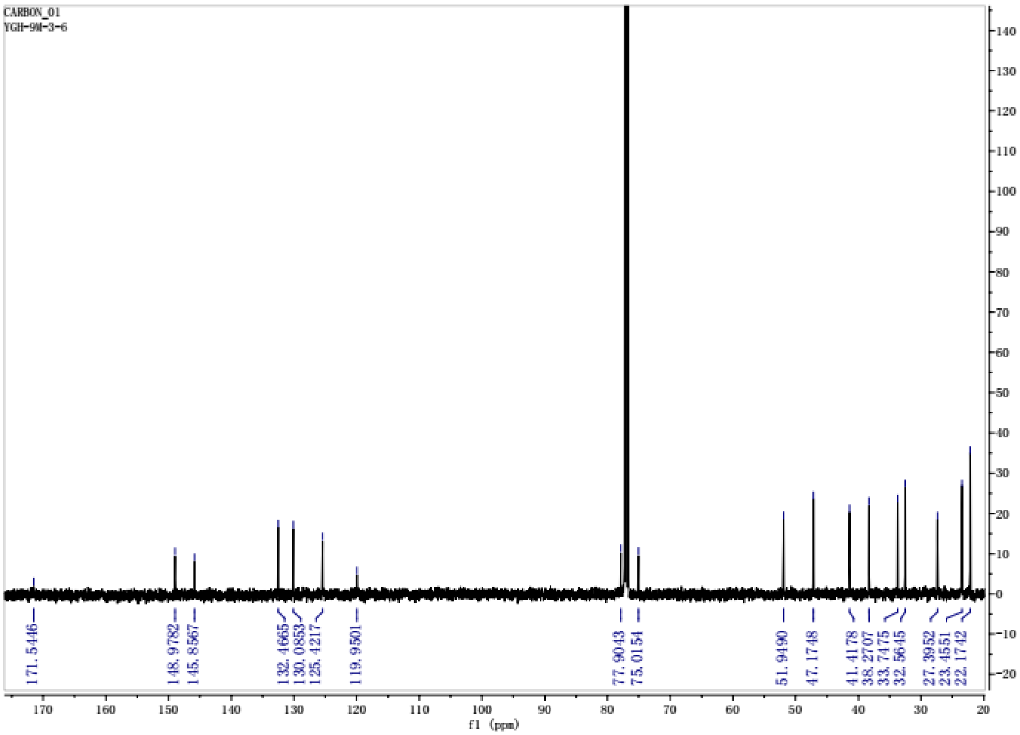

Figure S38. HMQC Spectrum of tanzawaic acid W (**6**)

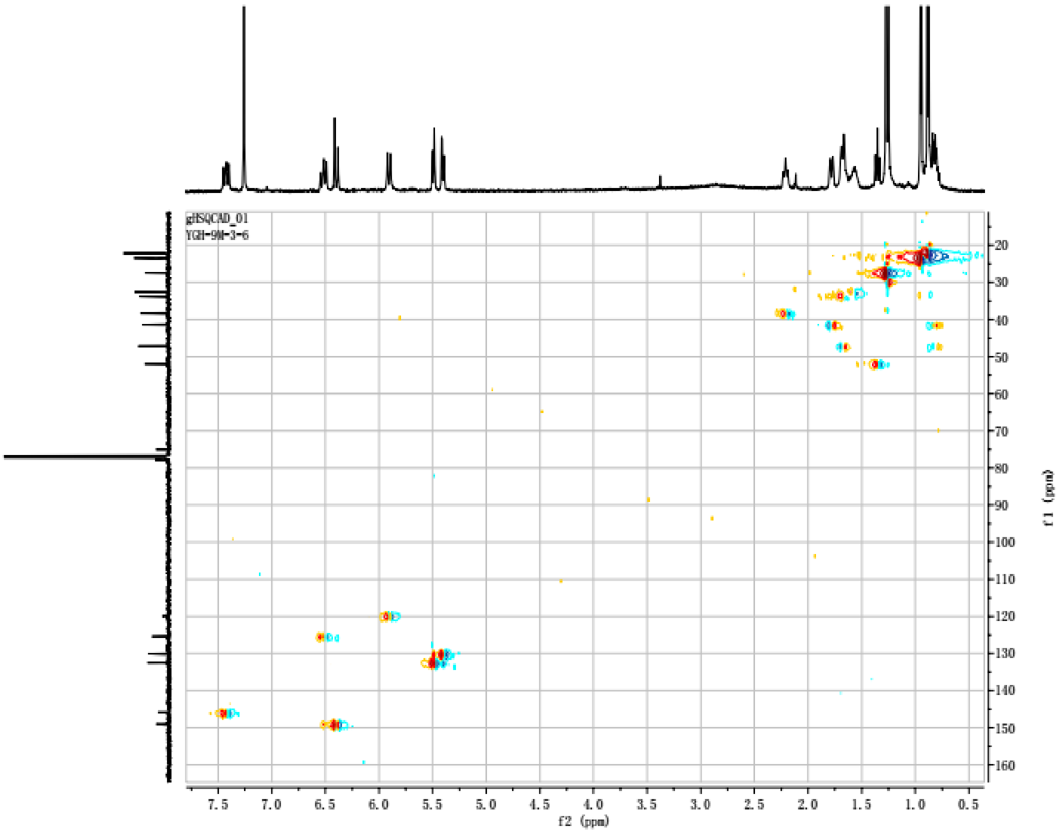

Figure S39. COSY Spectrum of tanzawaic acid W (**6**)

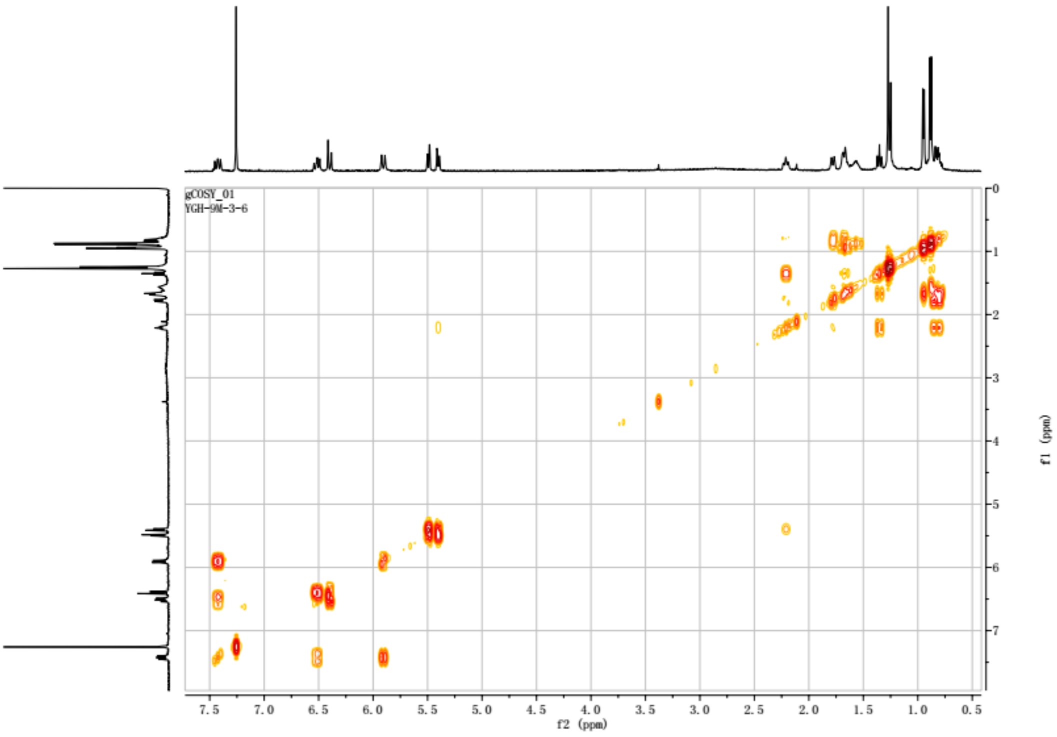

Figure S40. HMBC Spectrum of tanzawaic acid W (**6**)

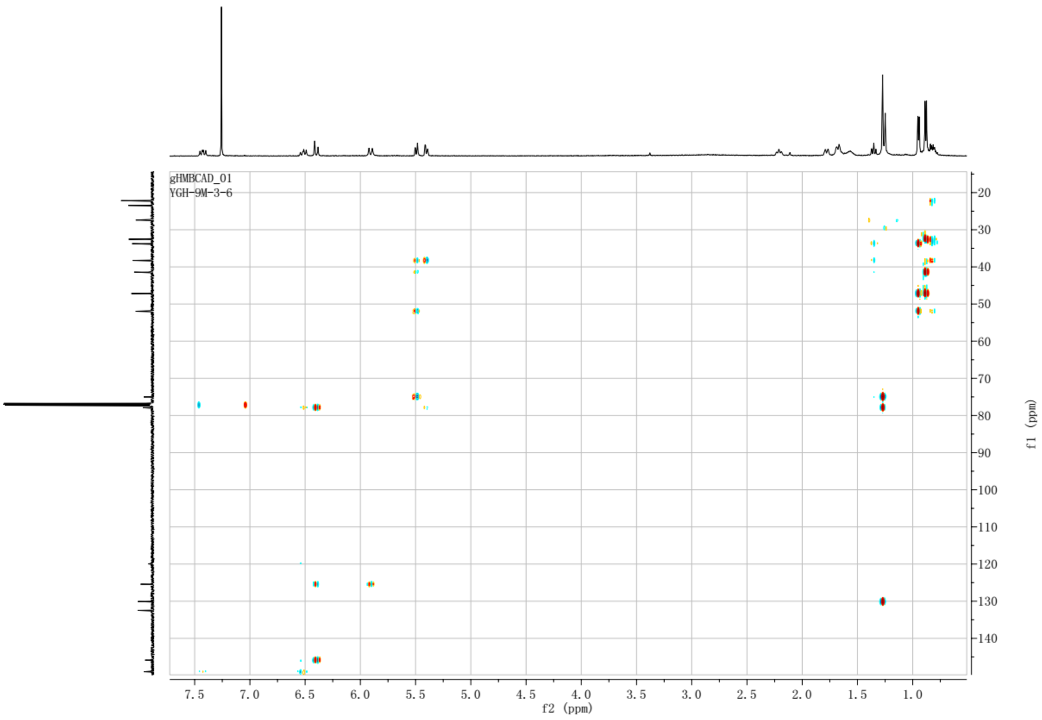

Figure S41. NOESY Spectrum of tanzawaic acid W (**6**)

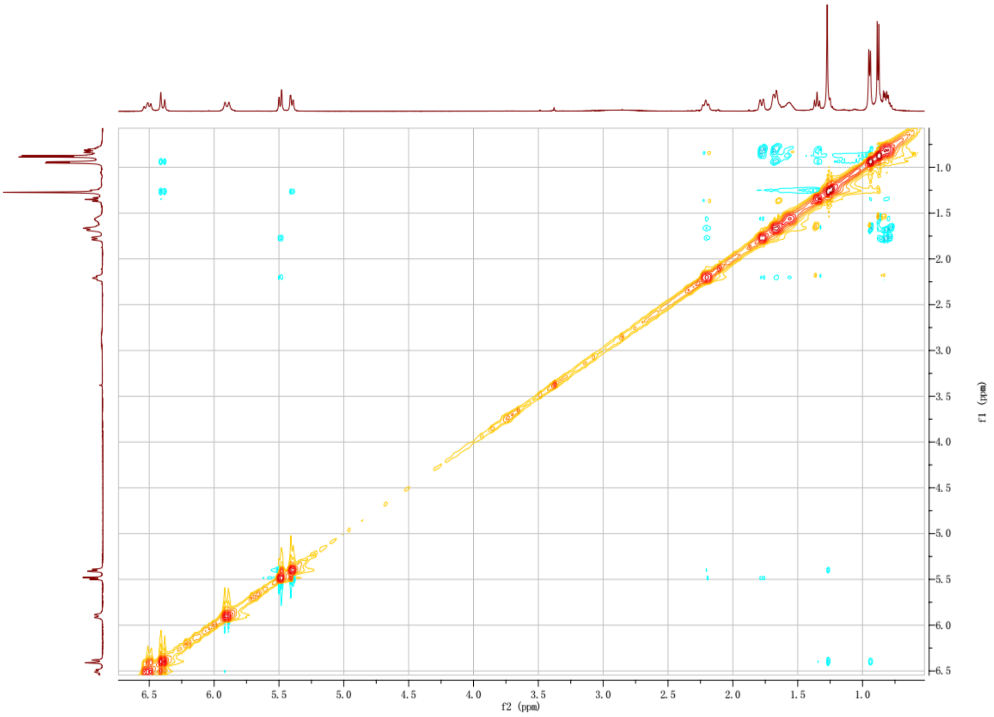

Figure S42. HRESIMS Spectrum of tanzawaic acid W (**6**)

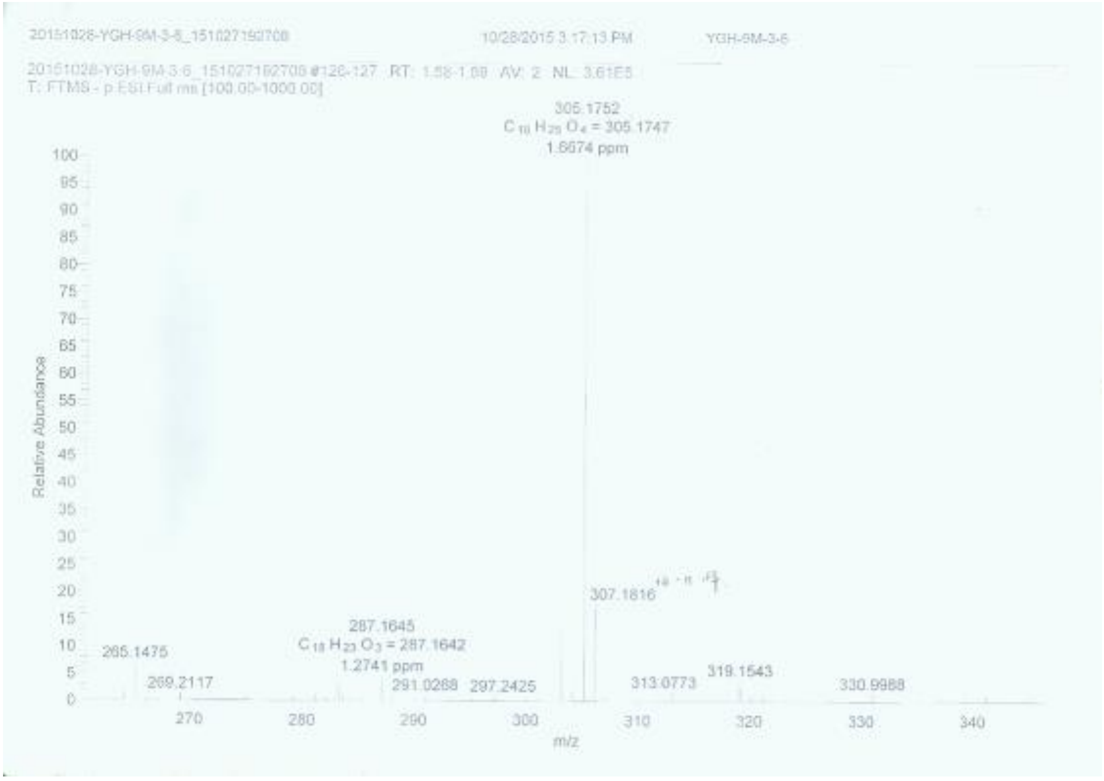

Figure S43. ^{1}H NMR Spectrum (500 MHz) of tanzawaic acid X (**11**) in $CDCl_3$

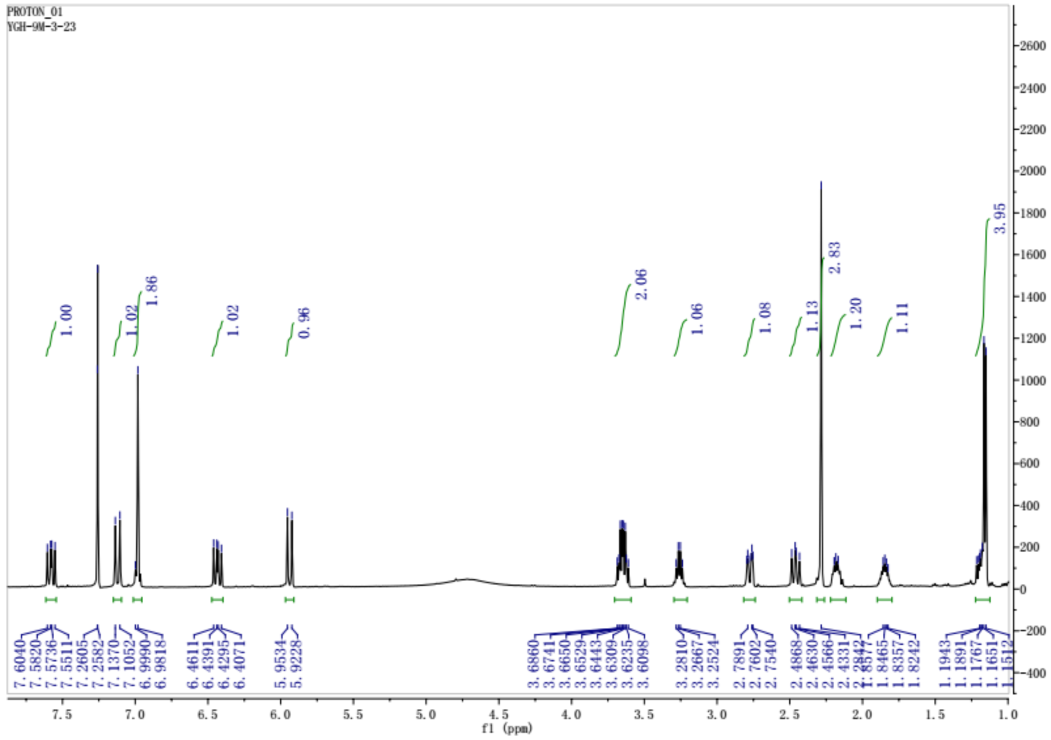

Figure S44. ^{13}C NMR Spectrum (125 MHz) of tanzawaic acid X (**11**) in $CDCl_3$

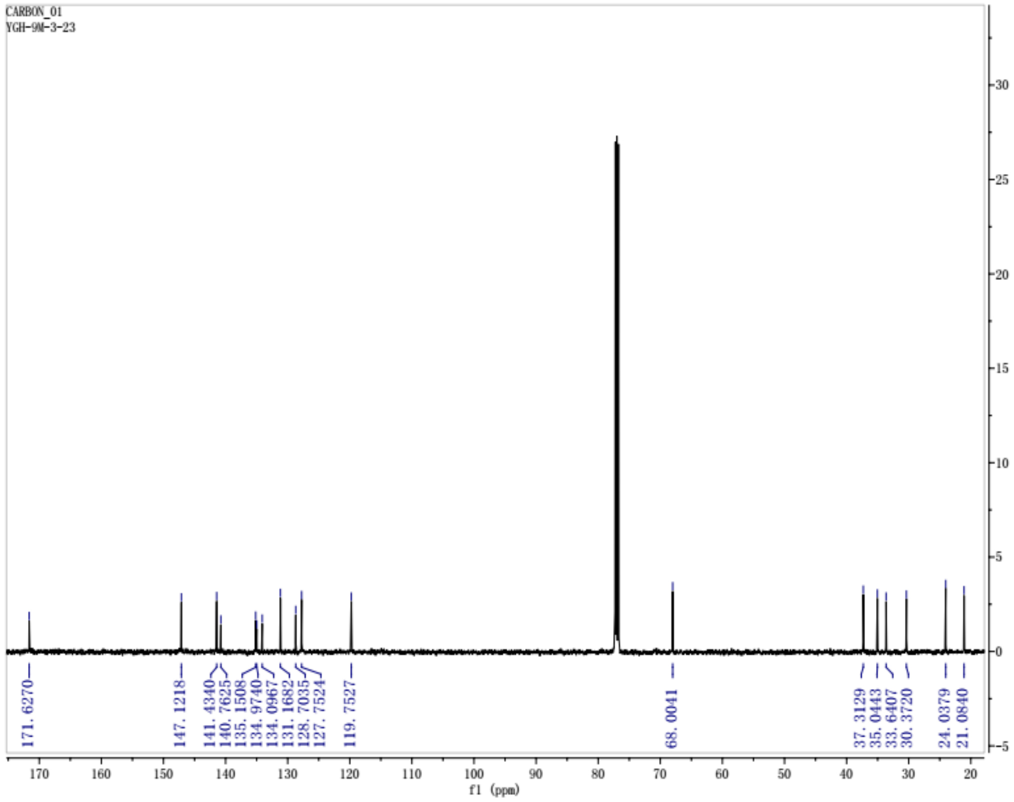

Figure S45. HMQC Spectrum of tanzawaic acid X (**11**)

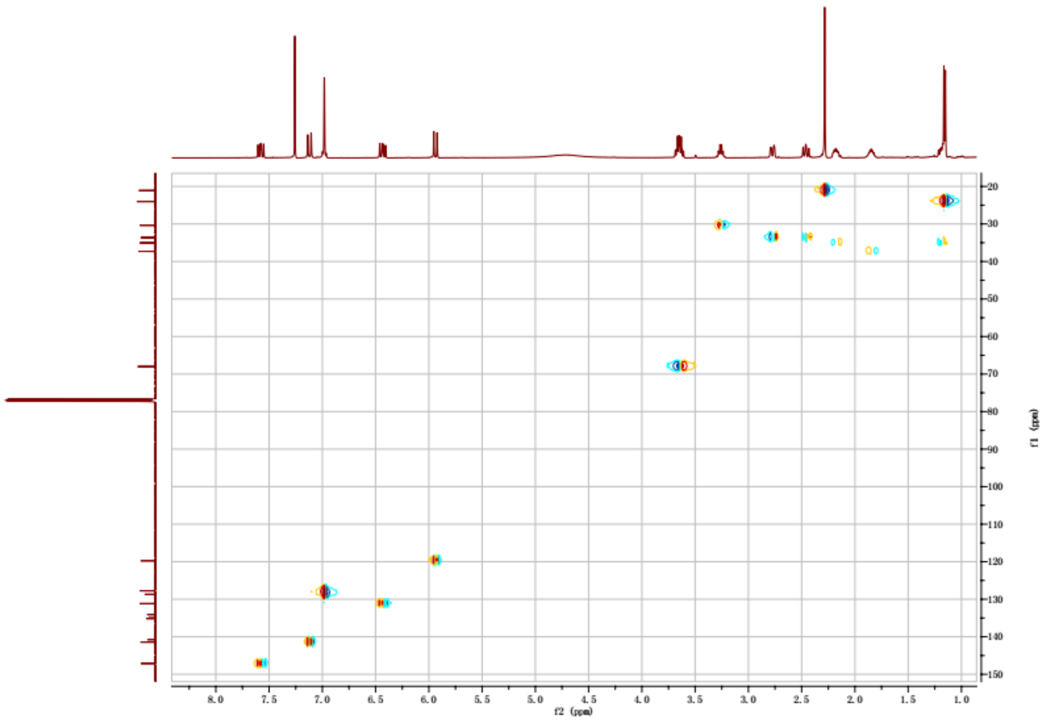

Figure S46. COSY Spectrum of tanzawaic acid X (**11**)

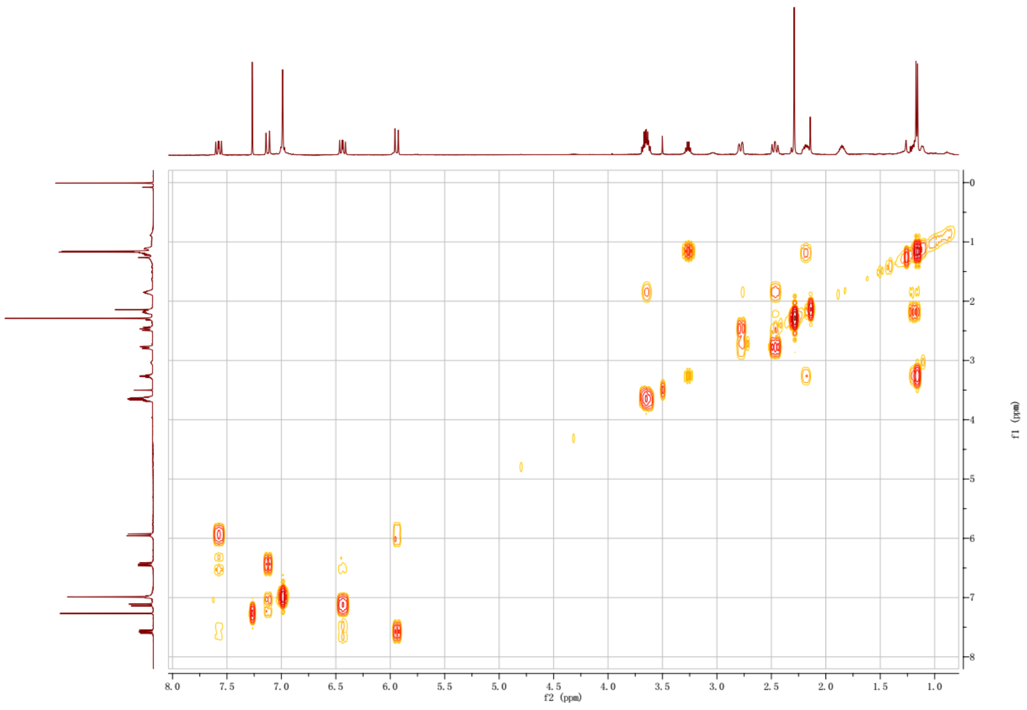

Figure S47. HMBC Spectrum of tanzawaic acid X (**11**)

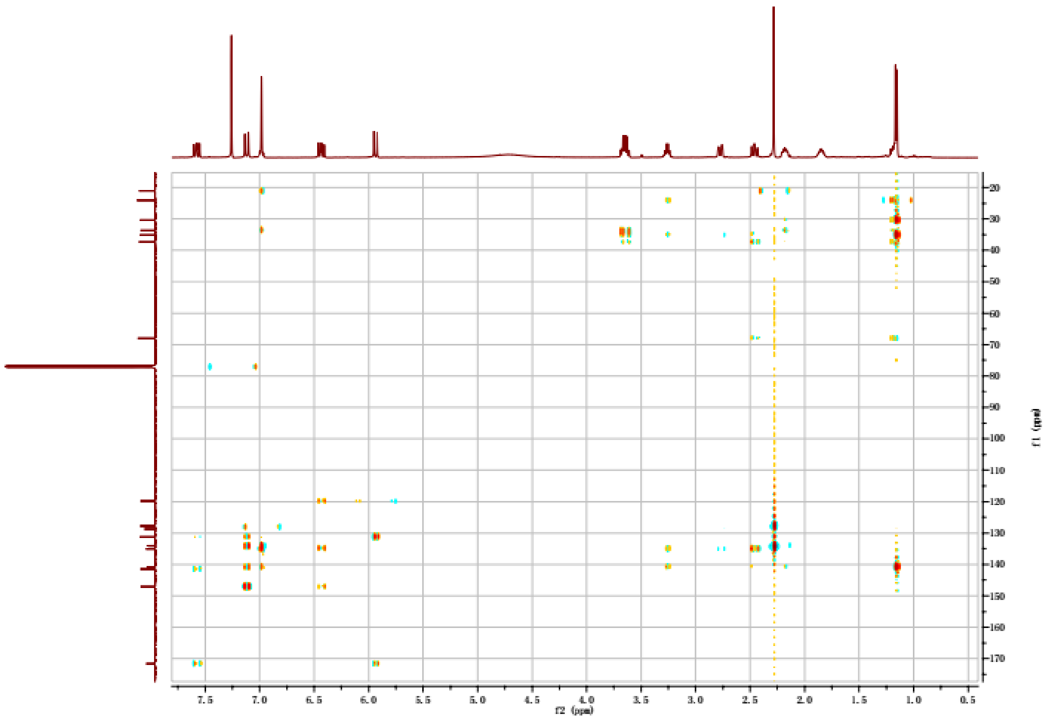

Figure S48. NOESY Spectrum of tanzawaic acid X (**11**)

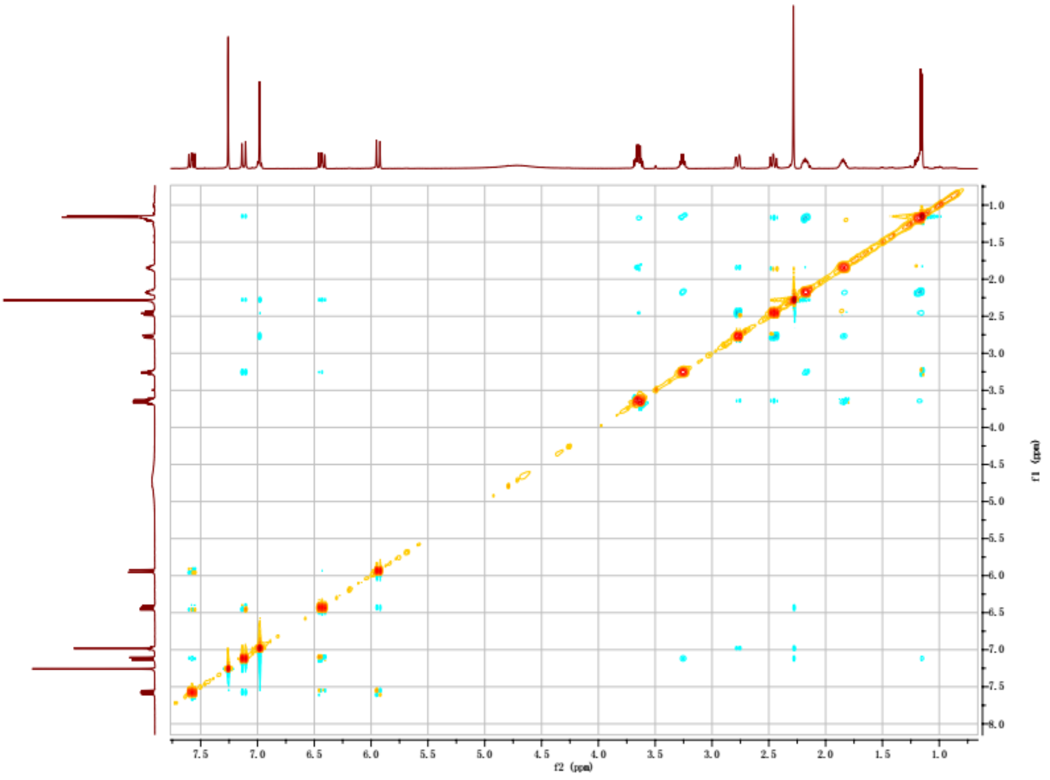

Figure S49. HRESIMS Spectrum of tanzawaic acid X (**11**)

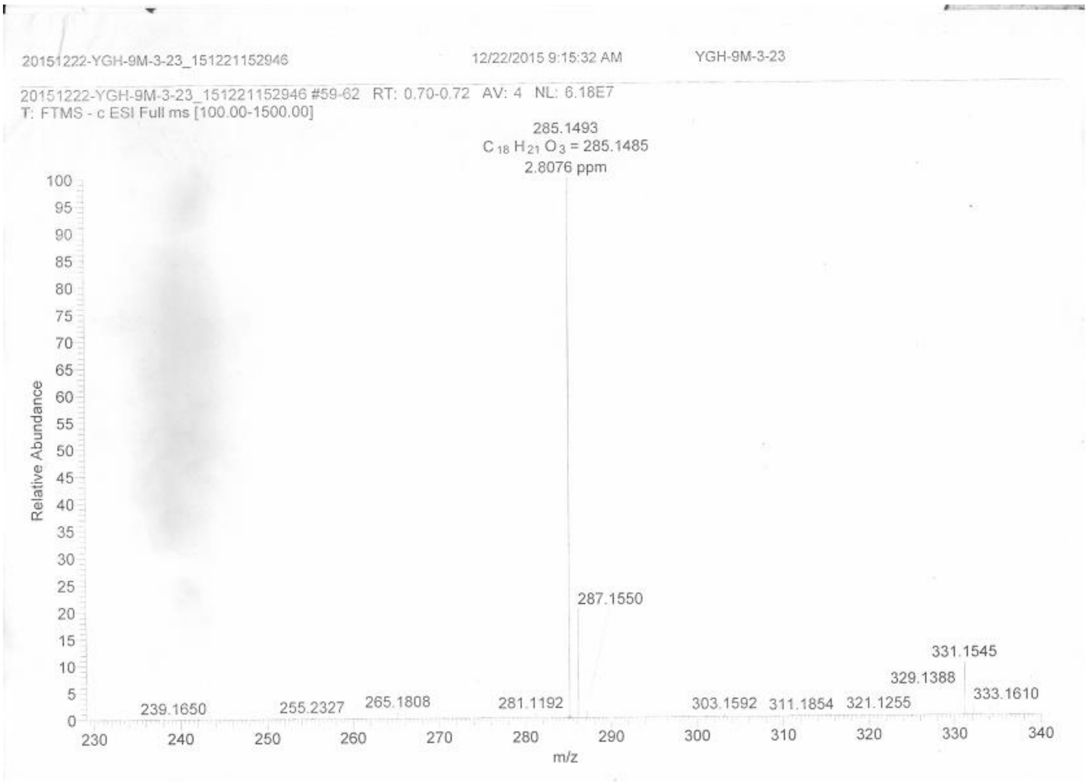

Figure S50. ^{1}H NMR Spectrum (500 MHz) of **1a** in Methanol-d_4

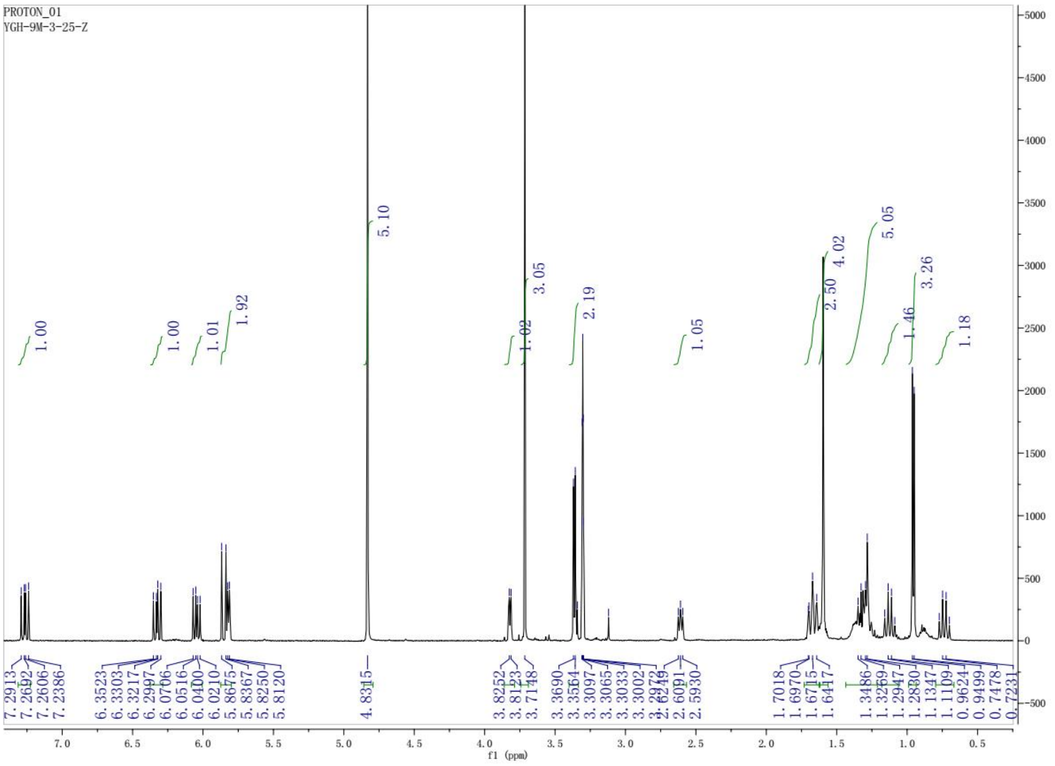

Figure S51. ^{1}H NMR Spectrum (500 MHz) of **1b** in Methanol-d_4

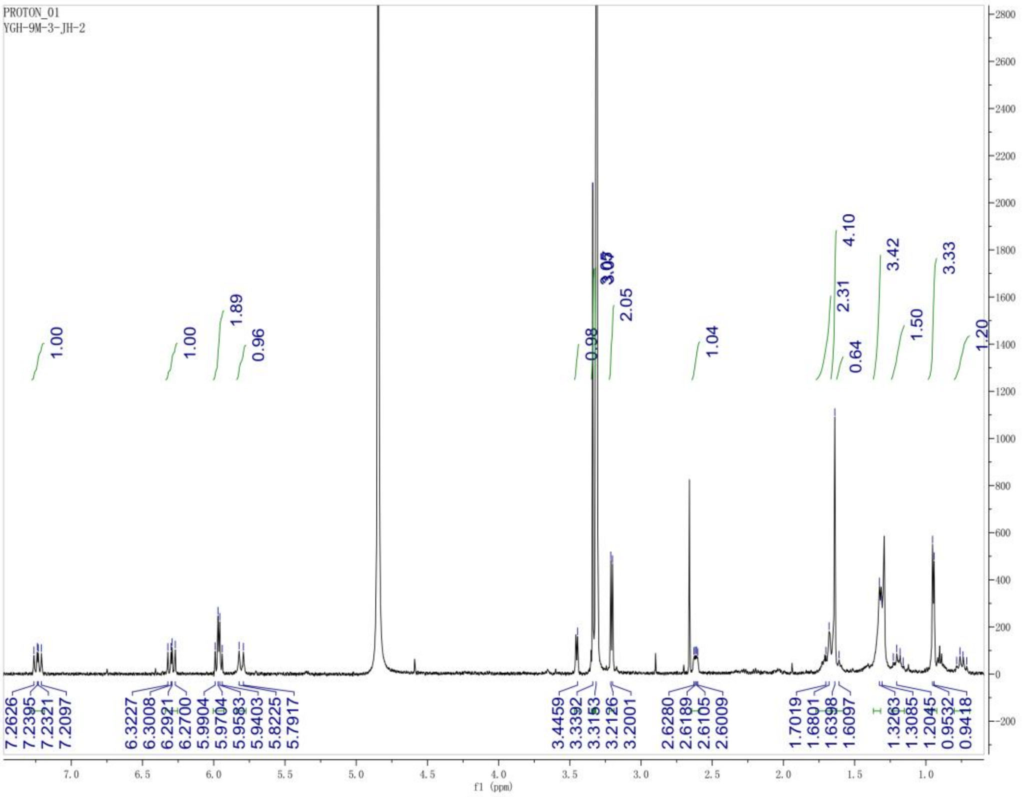

Figure S52. ^{13}C NMR Spectrum (125 MHz) of **1b** in Methanol-d_4

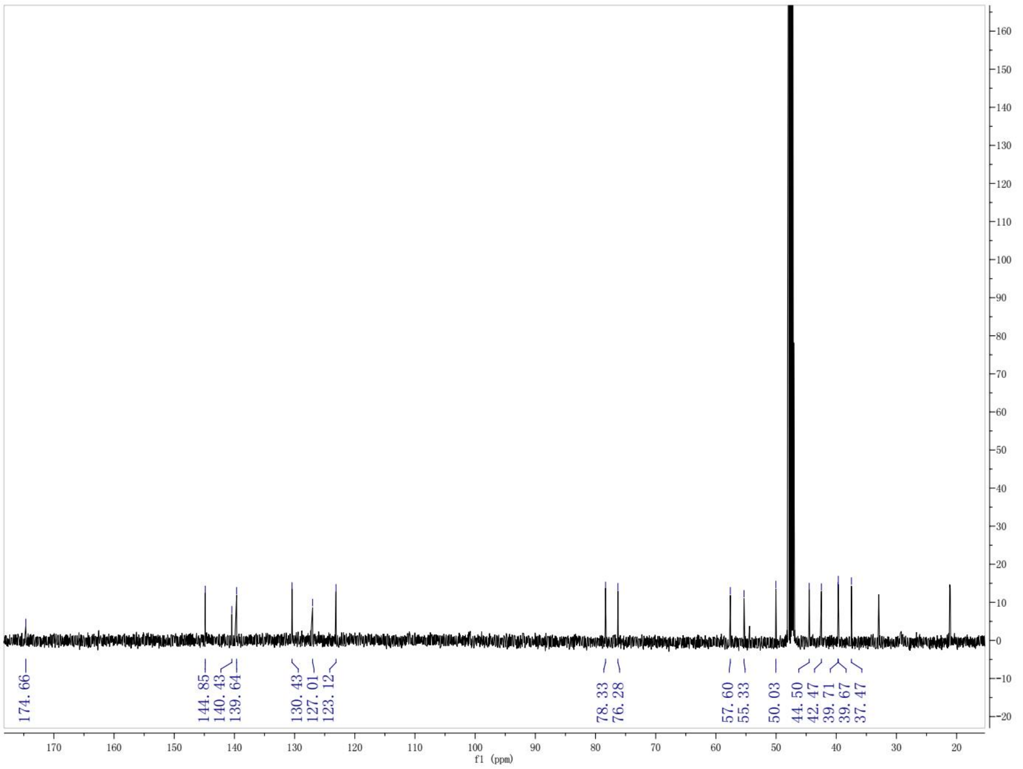

Figure S53. ^{1}H NMR Spectrum (500 MHz) of **12a** in $CDCl_3$

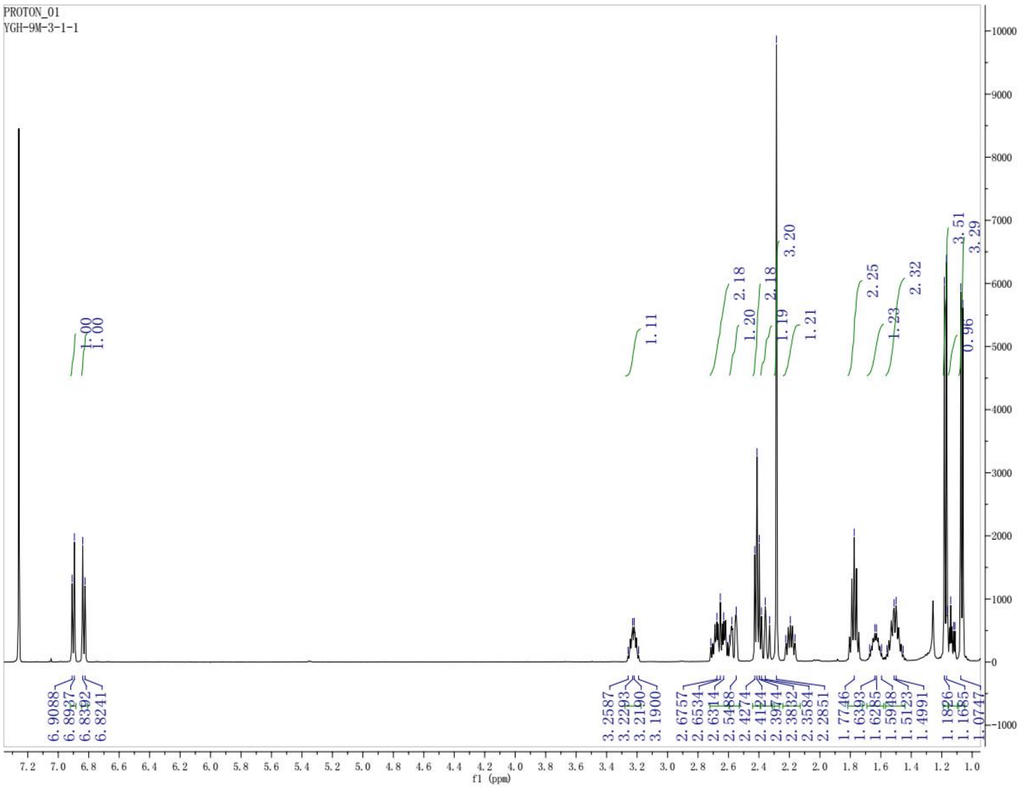

Figure S54. ^{13}C NMR Spectrum (125 MHz) of **12a** in $CDCl_3$

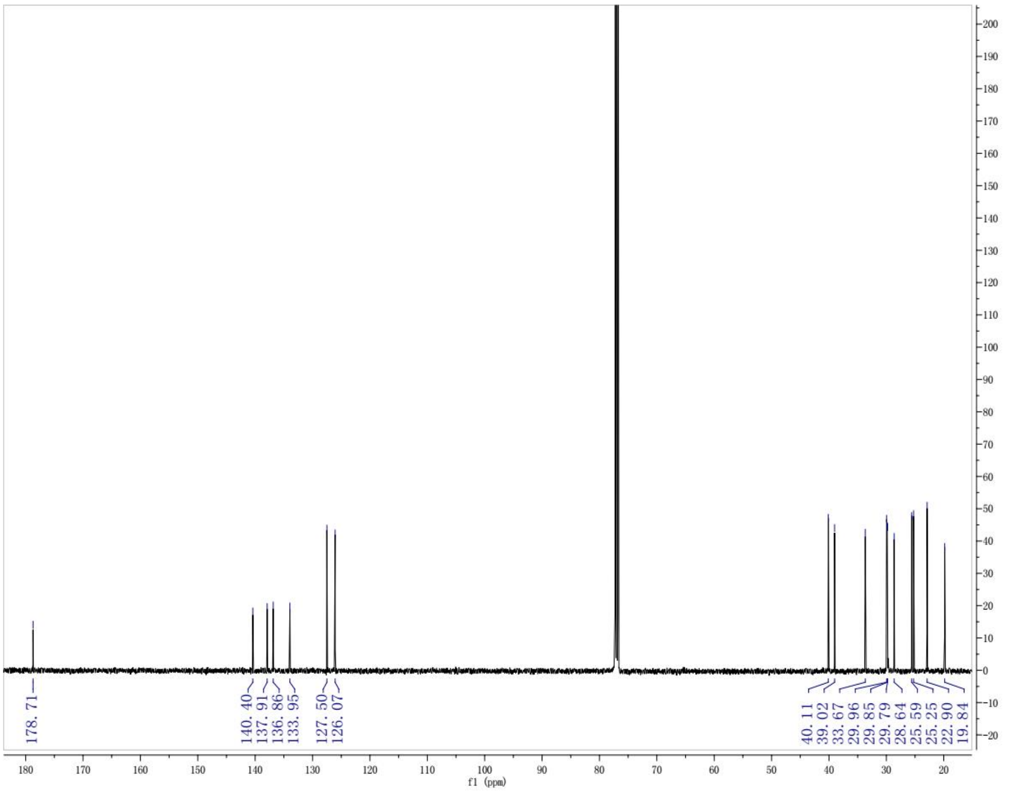

Figure S55. IR spectrum of tanzawaic acid R (**1**)

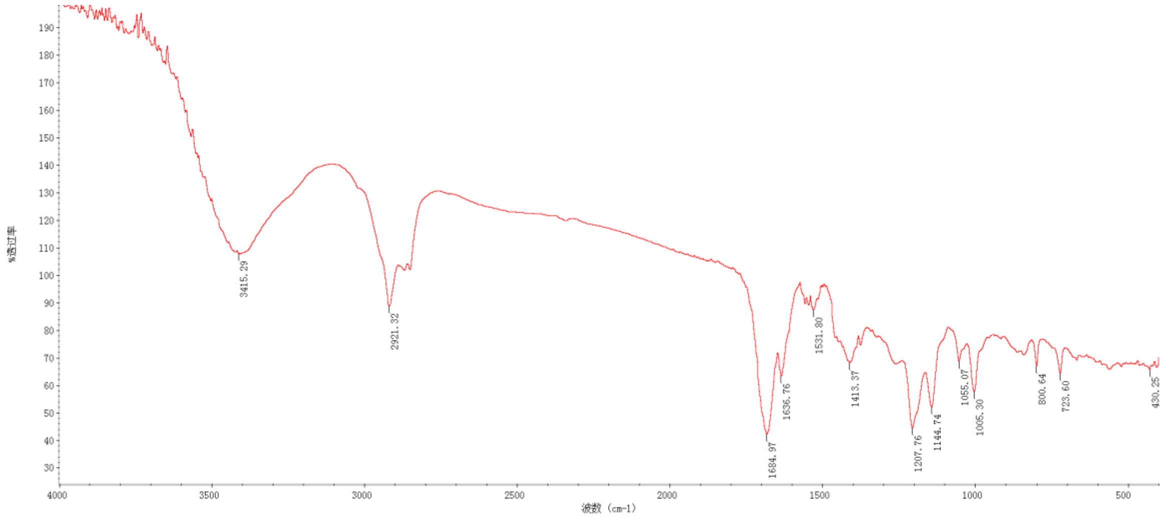

Figure S56. IR spectrum of tanzawaic acid S (**2**)

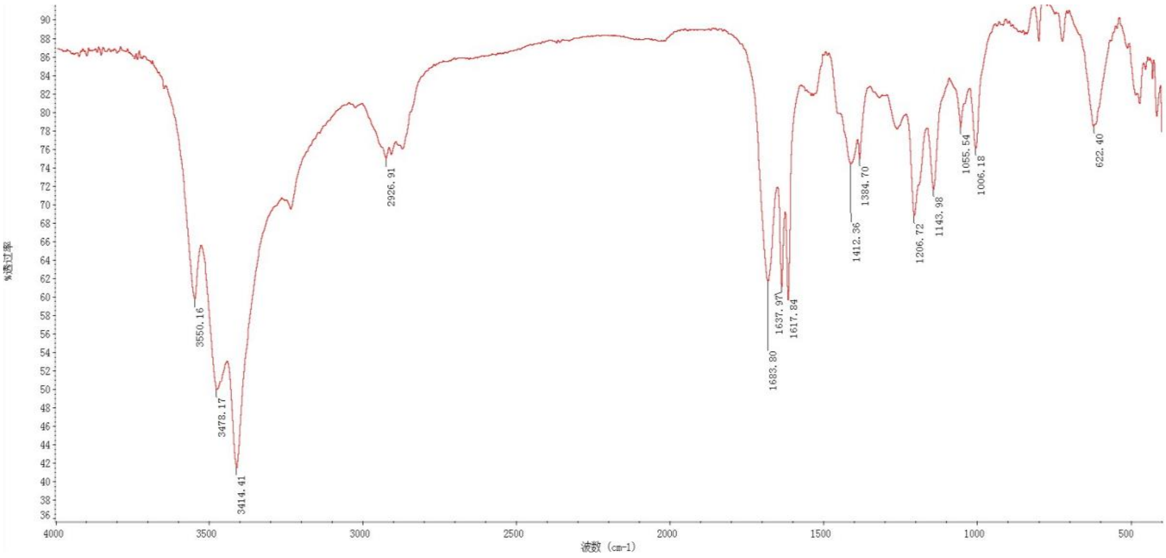

Figure S57. IR spectrum of tanzawaic acid T (**3**)

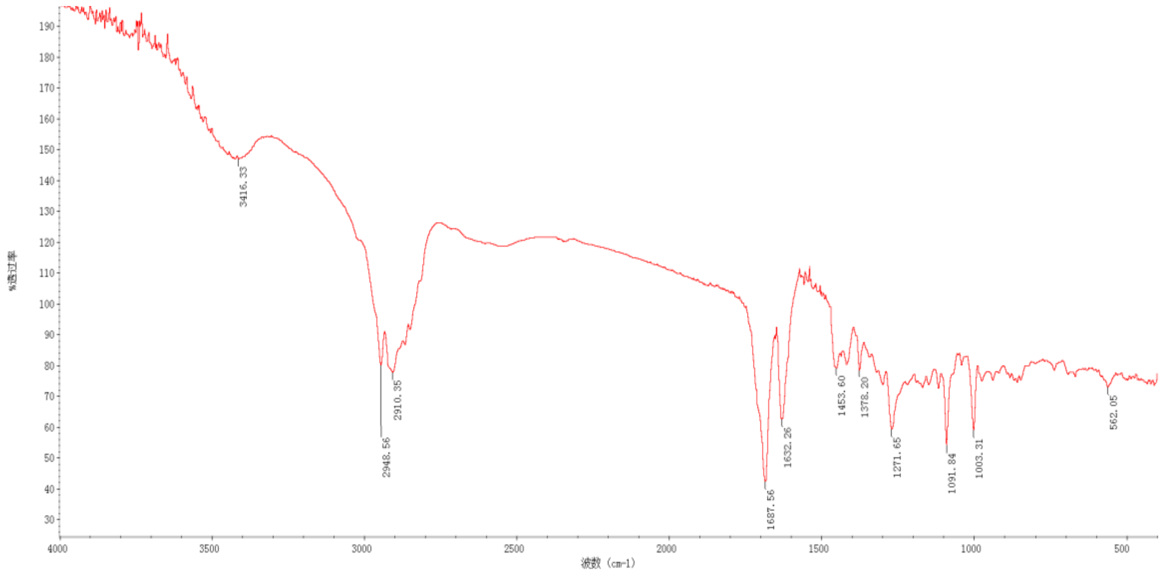

Figure S58. IR spectrum of tanzawaic acid U (**4**)

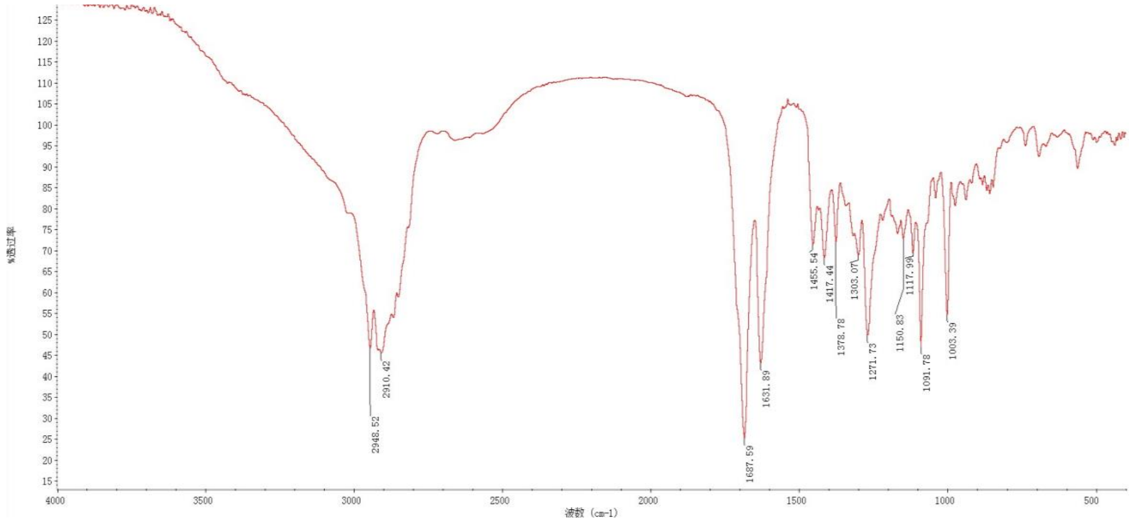

Figure S59. IR spectrum of tanzawaic acid V (**5**)

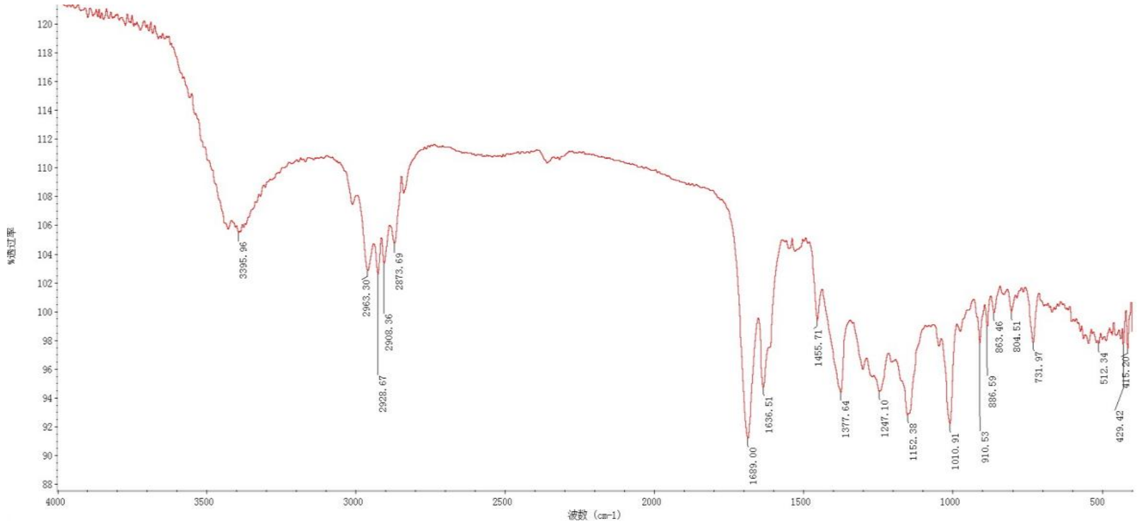

Figure S60. IR spectrum of tanzawaic acid W (**6**)

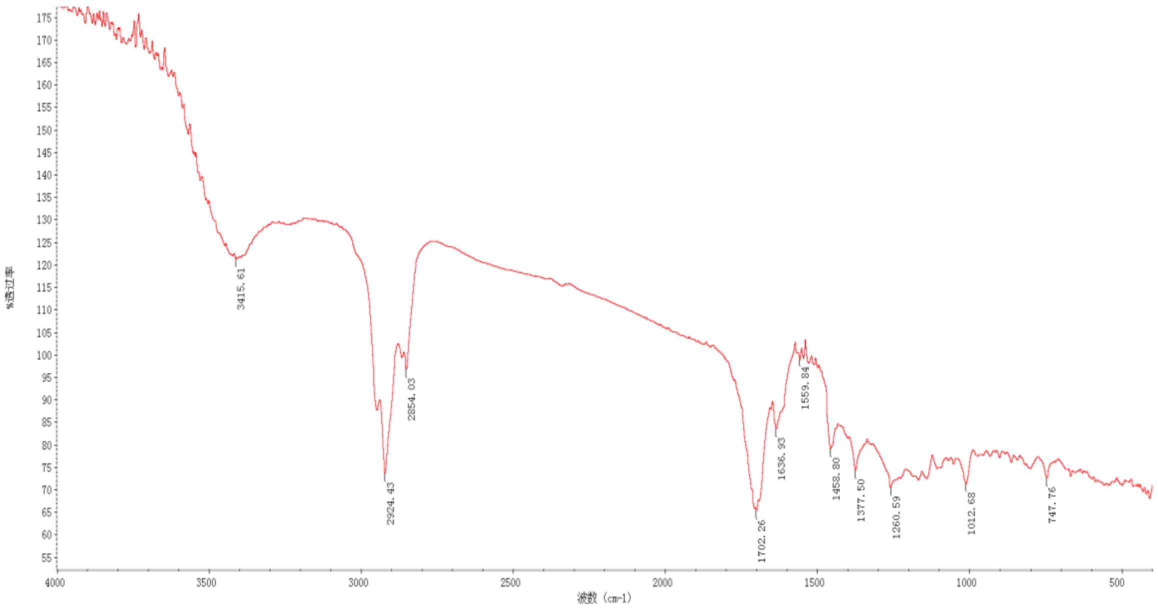

Figure S61. IR spectrum of tanzawaic acid X (**11**)

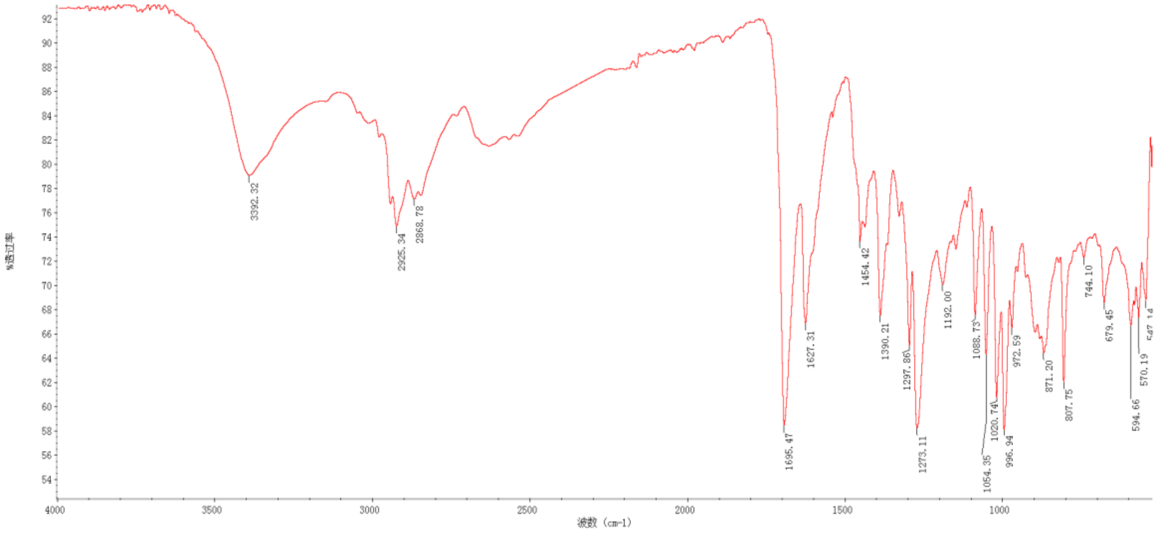

Supplement: Supplementary file 1 [file marinedrugs-16-00025-s001.pdf]
